# Digital Screen Time and Myopia: A Systematic Review and Dose-Response Meta-Analysis

**DOI:** 10.1001/jamanetworkopen.2024.60026

**Published:** 2025-02-21

**Authors:** Ahnul Ha, Yun Jeong Lee, Marvin Lee, Sung Ryul Shim, Young Kook Kim

**Affiliations:** 1 Department of Ophthalmology, Jeju National University Hospital, Jeju-si, Korea; 2 Department of Ophthalmology, Jeju National University School of Medicine, Jeju-si, Korea; 3 Department of Ophthalmology, Chungnam National University Hospital, Daejeon, Korea; 4 Allbarun Eye Clinic, Suwon-si, Korea; 5 Department of Biomedical Informatics, College of Medicine, Konyang University, Daejeon, Korea; 6 Ranelagh Center for Biosocial Informatics, Seoul National University College of Medicine, Seoul, Korea; 7 Department of Ophthalmology, Seoul National University Hospital, Seoul, Korea; 8 Trinity Biomedical Sciences Institute, School of Biochemistry and Immunology, Trinity College Dublin, The University of Dublin, Dublin, Ireland

## Abstract

**Importance:**

The association of digital screen time with myopia has been documented, but the dose-response association and safe exposure threshold remain unclear.

**Objective:**

To evaluate the dose-response association of time spent on digital screens with myopia risk.

**Data Sources:**

PubMed, EMBASE, Cochrane Library databases, CINAHL, and ClinicalTrials.gov were searched for full-length articles from peer-reviewed journals without restrictions on study design, publication date, or language from inception to November 25, 2024.

**Study Selection:**

Primary research articles investigating the association of exposure to digital screen devices (ie, smartphones, tablets, game consoles, computers, or television) with myopia-related outcomes (ie, prevalent or incident myopia and the rate of myopia progression) were identified by reviewers.

**Data Extraction and Synthesis:**

Two independent reviewers extracted data using a standardized procedure in accordance with the Meta-Analysis of Observational Studies in Epidemiology (MOOSE) and Preferred Reporting Items for Systematic Reviews and Meta-Analyses (PRISMA) reporting guidelines. A random-effects, dose-response meta-analysis (DRMA) was utilized to examine the pattern of the association of screen time with myopia.

**Main Outcome and Measures:**

Increased odds of myopia per hour of daily screen time.

**Results:**

In the linear DRMA of 45 studies with 335 524 participants (mean [SD] age, 9.3 [4.3] years), an additional hour of daily screen time was associated with higher odds of myopia (odds ratio [OR], 1.21; 95% CI, 1.13-1.30). The nonlinear DRMA of 34 studies with 314 910 participants also indicated higher odds of myopia with increasing screen time, ranging from 1 hour of daily exposure (OR, 1.05; 95% CI, 1.01-1.09) to 4 hours (OR, 1.97; 95% CI, 1.56-2.40). The dose-response curve showed myopia risk increasing significantly between 1 to 4 hours of daily screen time, and then rising more gradually after 4 hours.

**Conclusions and Relevance:**

In this systematic review and DRMA, a daily 1-hour increment in digital screen time was associated with 21% higher odds of myopia and the dose-response pattern exhibited a sigmoidal shape, indicating a potential safety threshold of less than 1 hour per day of exposure, with an increase in odds up to 4 hours. These findings can offer guidance to clinicians and researchers regarding myopia risk.

## Key Points

**Question:**

What is the dose-response association of digital screen time with the risk of myopia?

**Findings:**

This systematic review and dose-response meta-analysis of 45 studies involving 335 524 individuals revealed a significant dose-response association, characterized by a sigmoidal curve, of screen time with the odds of myopia. Myopia risk increased significantly from 1 to 4 hours of screen time and then rose more gradually thereafter.

**Meaning:**

These findings can offer guidance for clinicians and researchers and underscore the need to refine dose-response models for digital screen time and myopia risk to better address the myopia pandemic.

## Teaser

This systematic review and meta-analysis assesses associations of time spent on digital screens with the risk of myopia.

## Introduction

Myopia prevalence is on the rise, with projections suggesting that by 2050, nearly one-half of the world’s population will have it.^1,2^ This increase is coupled with earlier onset,^3^ faster progression,^4^ and greater severity of myopia at stabilization.^5^ It indicates, moreover, a future surge in the global burden of myopia-related sight-threatening conditions including macular degeneration, retinal detachment, and glaucoma.^6,7^

The projected surge in myopia cases is likely fueled by environmental factors prevalent in urbanized societies, with major contributors being increased near-vision activities and reduced outdoor time.^8,9,10^ The widespread adoption of digital devices over the past decade has introduced new forms of near-work activity. In research, digital screen time is typically defined as the duration of exposure to electronic displays including smartphones, tablets, gaming consoles, computers, and televisions, measured either for individual devices or as combined usage.^11^ As children increasingly embrace smart devices at younger ages and spend more time on digital screens, there is an urgent need to better understand the association of digital screen time with myopia.

A previous meta-analysis^12^ that pooled data from 11 studies investigating the association of screen time on smart devices (smartphones or tablets), either alone or combined with computer screen time, uncovered a significant association with myopia; smart device screen time alone was associated with a 26% increase in the odds of myopia, while in combination with computer use, the odds increased by 77%. In contrast, a recent meta-analysis^13^ that separately analyzed categorical and continuous screen time data found screen time on computers and televisions was associated with myopia, whereas smartphone use was not. Overall, the findings of the published studies in this field remain inconsistent. Moreover, the dose-response association of screen time with myopia has yet to be thoroughly investigated.

To address these gaps, we expanded the scope of previous meta-analyses both quantitatively and qualitatively by updating the pool of selected studies. Additionally, we conducted a dose-response meta-analysis (DRMA) to investigate the potential nonlinear association of digital screen time with myopia, with the aim of identifying a possible safety threshold for screen time exposure.

## Methods

The protocol for this systematic review and DRMA was prospectively registered on PROSPERO (CRD42024514134). This study was conducted in accordance with the Preferred Reporting Items for Systematic Reviews and Meta-Analyses (PRISMA) reporting guideline^14^ and the Meta-Analysis of Observational Studies in Epidemiology (MOOSE) reporting guideline.

### Search Strategy

We systematically searched the PubMed, Embase, Cochrane Library databases, CINAHL, and clinicaltrials.gov, and manually reviewed reference lists for studies on the association of digital screen device use with myopia from inception up to November 25, 2024. We included only studies published as full-length articles in peer-reviewed journals, without imposing any language or publication date restrictions. Two reviewers (A.H. and Y.K.K.) independently searched the literature and performed further cross-checking of the reference lists. Non–English-language reports were assessed by an individual fluent in the language.

Our search strategies were based on established terminology that included medical subject headings and EMBASE search terms, among which were *smartphone, screen time, cell phone, myopia, and refractive errors*. The terms had been selected to be broad enough to identify publications that had considered digital screen devices as one among many risk factors for myopia. The full search strategies are available in eAppendix 1 in Supplement 1.

### Selection Criteria

To identify relevant articles, the titles and abstracts of the retrieved papers were exported to Endnote version X9 (Thomson Reuters), where duplicates were removed. Then, 2 reviewers (A.H. and Y.K.K.) independently screened all titles and abstracts, thoroughly reviewing full texts for articles deemed potentially eligible based on the title and abstract content. Articles exploring risk factors for myopia were included even if digital screen devices were not explicitly mentioned in the title or in the abstract because digital screen device use might have been reported in the main text. We did not impose any baseline age or myopia status limitations on the study population. The exclusion criteria were articles not investigating myopia-related outcomes (ie, prevalence, incidence, and progression) and articles not exploring risk factors for myopia. Studies were excluded if they did not involve risk factor analysis with digital screen devices, such as mobile phones, tablets, game consoles, computers, and televisions, either separately or combined. Additionally, studies that incorporated other near-vision activities, such as reading nondigital books and writing, also were excluded from the final analysis. We did not exclude studies involving interventions such as orthokeratology lenses to slow myopia progression as long as the study had investigated the association of digital screen time with the presence or absence of myopia rather than its progression.

To investigate the dose-response association, we identified studies reporting exposure over a specific time unit (for instance, odds ratios [ORs] for myopia per each additional hour of screen time) or contrasted multiple groups with differing degrees of digital screen time (such as the ORs for myopia in groups with more than 2 hours of screen time compared with groups with less than 2 hours). Conflicts regarding inclusion were resolved through adjudication by a third reviewer (Y.J.L.).

### Data Extraction

The following data were extracted from each study: name of first author, publication year, study design, country of origin, sample size, age and sex of participants, definition and measurement of myopia (objective methods or self-reported myopia), type and extent of digital screen exposure, outcomes associated with myopia (including prevalence, incidence, and rate of progression), statistical correlations between digital screen time and myopia-related outcomes (including ORs, hazard ratios, risk ratios, prevalence ratios, β coefficients, and 95% CIs), and factors used for adjustment. In studies presenting results graphically, numerical values were extracted from the graphs using the measuring tool in Adobe Acrobat XI (Adobe Systems Inc).^15,16^ In studies where the 95% CI was not provided, values from studies with equivalent sample sizes were adopted.^17^ Adjusted data were included when available; otherwise, unadjusted data were used. Two investigators (A.H. and Y.K.K.) independently extracted data, which were then input into Microsoft Access 2021 (Microsoft Corporation). Conflicting data entries were identified by algorithmic methods and resolved through discussion.

### Bias Assessment and Overall Quality of Evidence

The risk of bias of the included studies was assessed using the Newcastle-Ottawa Scale, rated by 2 independent reviewers (A.H. and Y.J.L.) with disputes resolved by a third independent reviewer (M.L.), as detailed in eAppendix 2 in Supplement 1.^18^ Publication bias was assessed qualitatively with a funnel plot^19,20^ and quantitatively with an Egger test,^21^ a statistical equivalent of the funnel plot. The overall quality of evidence was determined using the Grading of Recommendations, Assessment, Development, and Evaluations (GRADE) framework.^22^

### Statistical Analysis

Transformations performed to facilitate inclusion of results in the meta-analysis were conversion of β coefficients to ORs and standardization of ORs associated with digital screen time from minutes per day to hours per day (eMethods in Supplement 1).^23,24^ When studies provided ORs for overlapping samples (eg, weekday vs weekend use), we selected ORs with larger exposed samples and longer data collection periods (eg, weekdays).

We implemented a single-stage, random-effects meta-analysis of the dose-response model using the dosresmet package in R version 4.0.3 (R Project for Statistical Computing).^25,26^ To illustrate the linear dose-response model, we computed the OR and its corresponding 95% CI for myopia per additional hour of daily screen time in each individual study. Subsequently, we combined the study-specific ORs to estimate the pooled OR along with its 95% CI using a random-effects model implemented through the metafor package in R. To illustrate the nonlinear dose-response model, we followed the established DRMA guidelines, placing knots at the 5th, 35th, 65th, and 95th percentiles of the exposure distribution. This method ensures a balanced data distribution and provides flexibility to capture nonlinearity without overfitting; furthermore, it is widely recognized for its robust performance in previous DRMAs.^6,27^

To assess the robustness of our main findings, we conducted subgroup analyses based on the following hypotheses, assuming a greater OR for myopia for (1) prevalence compared with incidence or progression assessments in myopia-related outcomes, (2) cross-sectional studies compared with cohort or longitudinal study designs, (3) studies including participants aged 19 years and older compared with those aged 2 to 7 years or 8 to 18 years, (4) studies conducted in Asia compared with regions outside of Asia, and (5) studies examining combined digital devices compared with single devices. Additionally, we specifically analyzed data from studies with more than 500 participants, where adjusted data were used, myopia was confirmed by cycloplegic refraction, and the association of smartphone screen time with myopia was exclusively investigated. For the nonlinear dose-response model, sensitivity analyses were conducted by (1) adjusting knot numbers at different doses, (2) analyzing data specifically from studies with more than 500 participants, (3) including only studies where myopia was confirmed by cycloplegic refraction, and (4) restricting the analysis to participants younger than 19 years. Statistical significance was considered a 2-sided *P* < .05.

## Results

### Search Results and Characteristics of Included Studies

Our systematic search process is shown in eFigure 1 in Supplement 1. The final analysis included 45 studies^23,28,29,30,31,32,33,34,35,36,37,38,39,40,41,42,43,44,45,46,47,48,49,50,51,52,53,54,55,56,57,58,59,60,61,62,63,64,65,66,67,68,69,70,71^ (eAppendix 3 in Supplement 1), with details of excluded articles provided in eAppendix 4 in Supplement 1. The results of the Newcastle-Ottawa Scale assessment are presented in eTable 1 in Supplement 1. Among the 45 studies included (Table 1), with a total study population of 335 524 individuals (mean [SD] age 9.3 [4.3] years), 33 studies defined myopia based on spherical equivalent,^28,29,30,31,32,33,34,35,36,37,38,39,40,41,42,43,44,45,46,47,48,49,50,51,52,53,54,55,56,57,58,59,60^ while 12 relied on self-reported questionnaires.^23,61,62,63,64,65,66,67,68,69,70,71^

**Table 1.** Characteristics of Studies Included in the Meta-Analysis Examining the Dose-Response Association of Digital Screen Time With Myopia

Source	Myopia prevalence, %	Study design	Participants, No.	Age, mean (SD), y	Country	Myopia definition (measure)	Screen exposure	Myopia related outcome	Association of screen exposure with myopia, OR (95% CI)	Adjusted factors
Damian et al,^54^ 2010^a^	12.4	Cross-sectional	5865 Children and adolescents	11.9 (3.3)	Poland	SE ≤−0.5 D (cycloplegic refraction)	Computer or television	Prevalence	Percentage of myopia prevalence: <0.8 h/d, 11.45%; >0.8 h/d, 13.85%	None
Saxena et al,^28^ 2015^a^^,^^b^	13.1	Cross-sectional	9884 Children and adolescents	11.6 (2.2)	India	SE ≤−0.5 D in either or both eyes (cycloplegic AR)	Computer, video, or mobile games	Prevalence	0 h/wk, 1 [Reference]; 1-4 h/wk, 4.50 (2.33 to 8.98); >4 h/wk, 8.10 (4.05 to 16.21)	Age, sex, type of school, family history of glasses, mother’s education, socioeconomic status, No. of h/wk reading/writing at school and home, No. of h/wk watching television, and No. of h/wk playing outdoor games
Chua et al,^29^ 2015^b^	6.1	Cohort	572 Children	3 (NR)	Singapore	SE <−0.5 D in right eye (cycloplegic AR)	Handheld digital device or computer	Incidence	Handheld device: 1.04 (0.67 to 1.61); Computer:0.92 (0.31 to 2.74)	Age, sex, ethnicity, maternal education level, and parental myopia
Schuster et al,^61^ 2017a^a^^,^^b^^,^^c^	13.3	Cross-sectional	12 884 Children	Range, 3-10 y	Germany	Self-reported (questionnaire)	Smartphone	Prevalence	<0.5 h/d, 1 [Reference]; 1-2 h/d, 1.13 (0.84 to 1.52); >2 h/d, 1.23 (0.86 to 1.76)	Age, sex, socioeconomic status (based on reported occupation, education, and parental income), and migration background
Schuster et al,^61^ 2017b^a^^,^^b^^,^^c^	13.3	Cross-sectional	12 884 Children and adolescents	Range, 11-17 y	Germany	Self-reported (questionnaire)	Smartphone	Prevalence	<0.5 h/d, 1 [Reference]; 1-2 h/d, 1.10 (0.78 to 1.53); >2 h/d, 1.36 (0.96 to 1.92)	Age, sex, socioeconomic status (based on reported occupation, education, and parental income), and migration background
Hagen et al,^30^ 2018^b^	13.4	Cross-sectional	439 School-aged adolescents	16.7 (0.9)	Norway	SE <−0.5 D (cycloplegic AR)	Smartphone, tablet, and computer	Prevalence	1.01 (0.78 to 1.31)	Sex
Guan et al,^31^ 2019^a^^,^^b^	77.4	Cross-sectional	19 934 Primary school–aged children	10.6 (1.2)	China	SE ≤−0.5 D in at least 1 eye (VA and (cycloplegic AR)	Smartphone and computer	Prevalence	Computer: 0 min/d, 0 [Reference]; 1-30 min/d, β = 0.017 (95% CI, −0.097 to 0.131); 31-60 min/d, β = 0.305 (95% CI, 0.141 to 0.468); >60 min/d, β = 0.032 (95% CI, −0.161 to 0.226); smartphone: 0 min/d, 0 [Reference]; 1-30 min/d, β = 0.025 (95% CI, −0.065 to 0.115); 31-60 min/d, β = −0.015 (95% CI, −0.215 to 0.185); >60 min/d, β = 0.161 (95% CI, −0.068 to 0.391)	Age, sex, family wealth, parental migrant status, parental education, and child’s residence
											Harrington et al,^32^ 2019^a^^,^^b^	14.3	Cross-sectional	1626 School-aged children and adolescents	Range, 6-7 y and 12-13 y	Ireland	SE ≤−0.5 D in either eye (cycloplegic AR)	Smartphone	Prevalence	<1 h/d, 0.3 (0.2 to 0.5); 1-3 h/d, 0.5 (0.3 to 0.8); >3 h/d, 1 [Reference]	Age, ethnicity, after-school activities, reading/writing in leisure time, daylight exposure during summer, birth season, duration of breastfeeding, BMI, and parental myopia
Huang et al,^62^ 2019^a^^,^^d^	86.8	Cross-sectional	968 First-year university students (young adults)	19.6 (0.9)	China	Self-reported (questionnaire)	Smartphone and computer	Prevalence	Computer: 0 h/d, 1 [Reference]; ≤1 h/d, 1.71 (0.90 to 3.26); 1.01-2 h/d, 1.38 (0.78 to 2.43); 2.01-3 h/d, 1.24 (0.70 to 2.20); >3 h/d, 0.73 (0.42 to 1.27); smartphone: 0 h/d, 1 [Reference]; ≤1 h/d, 0.78 (0.36 to 1.69); 1.01-2 h/d, 1.01 (0.47 to 2.18); 2.01-3 h/d, 0.72 (0.36 to 1.46); >3 h/d, 0.63 (0.33 to 1.20)	None
Alvarez-Peregrina et al,^33^ 2019	19.1	Cross-sectional	5441 School-aged children	6.2 (0.8)	Spain	SE <− 0.5 D (objective and subjective refraction)	Smartphone, tablet, and video game	Prevalence	Percentage of myopia prevalence: <25% of time in near activities, 24%; 25% to 50% of time, 23%; >50% of time, 53%	NR
Singh et al,^34^ 2019^a^^,^^b^	21.1	Cross-sectional	1234 School-aged children and adolescents	10.5 (3.0)	India	SE ≤−0.5 D in either or both eyes (cycloplegic refraction)	Smartphone and video game	Prevalence	0-2 h/d, 1 [Reference]; >2 to 4 h/d, 8.33 (3.54 to 19.58)	Age, sex, family history, outdoor play hours, and study hours (reading/writing)
											Liu et al,^35^ 2019^b^	59.2	Cross-sectional	566 Primary and secondary school–aged children	9.5 (2.1)	China	SE ≤−0.5 D in right eye (cycloplegic AR)	Smartphone and tablet	Prevalence	Smartphone: 0.90 (0.57 to 1.43); tablet: 1.40 (0.86 to 2.28)	Age, sex, BMI, monthly family income, parental myopia, time spent outdoors, time spent reading/writing, reading/writing distance, and daily sleep duration
Toh et al,^63^ 2019^b^	83.0	Cross-sectional	1884 Children, adolescents, and young adults	Range, 10-18 y	Singapore	Self-reported (questionnaire)	Smartphone and tablet	Prevalence	Smartphone: 0.97 (0.94 to 0.99); tablet: 0.99 (0.94 to 1.05)	Sex, school level, DASS-21, PAQ-A, and total technology use of other devices
Yang et al,^64^ 2020^a^	2.3	Cross-sectional	26 433 Preschool- and school-aged children	Range, 2-7 y	China	Self-reported (questionnaire)	Smartphone, tablet, or other handheld electronic screens	Prevalence	Percentage of myopia prevalence: <60 min/d, 1.77%; 60-120 min/d, 2.04%; >120 min/d, 3.84%	Age, sex, feeding patterns, premature birth, parental age at childbirth, education level, and monthly household income
Hansen et al,^36^ 2020^a^^,^^b^	25.0	Cohort	1443 Adolescents	Median (IQR), 16.6 (0.3) y	Denmark	SE ≤−0.5 D in right eye (subjective and objective refraction)	Smartphone, tablet, and computer	Prevalence	<2 h/d on weekdays, 1 [Reference]; 2-4 h/d, 1.89 (1.09 to 3.28); 4-6 h/d, 1.68 (0.98 to 2.89); >6 h/d, 1.89 (1.10 to 3.24)	Age, sex, weight, height, and physical activity
Schuster et al,^65^ 2020^a^^,^^b^	11.4	Cross-sectional	12 826 children and adolescents (wave 2)	9.20 (4.81)	Germany	Self-reported (questionnaire)	Game console and computer	Prevalence	Game console: none, 1 [Reference]; <1 h/d, 1.17 (0.98 to 1.40); 1-2 h/d, 1.06 (0.82 to 1.38); >2 h/d, 1.07 (0.79 to 1.45); computer: none, 1 [Reference]; <1 h/d, 1.00 (0.77 to 1.29); 1-2 h/d, 0.97 (0.71 to 1.32); >2 h/d, 1.05 (0.78 to 1.43)	Age, sex, socioeconomic status (occupation, education, and parental income), migration background, and all types of media use
Enthoven et al,^37^ 2020^a^^,^^d^	11.5	Cohort	5074 children	9.78 (0.34)	Netherlands	SE ≤−0.5 D in at least 1 eye (cycloplegic AR)	Computer	Prevalence	<5 h/wk, 1 [Reference]; 5-10 h/wk, 1.004 (0.981 to 1.027); >10 h/wk, 1.004 (0.974 to 1.034)	None
McCrann et al,^23^ 2020^b^	34.0	Cross-sectional	402 Students (adolescents and young adults)	16.8 (4.4)	Ireland	Self-reported (questionnaire)	Smartphone	Prevalence	1.026 (1.001 to 1.051)	Age, sex, No. of parents with myopia, and belief that technology negatively impacts eyes
Liu et al,^66^ 2021^b^	39.2	Cross-sectional	3918 Primary, secondary, and university students	NR	China	Self-reported (questionnaire)	Digital device (smartphone, computer, or television)	Prevalence	1.25 (1.21 to 1.30)	Age, sex, location of residence, and pre–COVID-19 pandemic myopia condition
Liu et al,^67^ 2021^b^	39.9	Cross-sectional	3405 Primary, lower secondary, and upper secondary school students	NR	China	Self-reported (questionnaire)	Electronic learning devices including smartphone, computer, and television	Prevalence	1.074 (1.058 to 1.089)	Age, sex, and location of residence
Liu et al,^68^ 2021^b^	36.3	Cross-sectional	3831 Preprimary, primary, lower secondary, or upper secondary school students	NR	China	Self-reported (questionnaire)	Digital device (smartphone, computer, and television)	Progression	1.30 (1.22 to 1.38)	Age, sex, location of residence, and prepandemic myopia condition
Enthoven et al,^38^ 2021^a^^,^^e^	18.9	Cohort	525 Children and adolescents	Range, 12-16 y	Netherlands	SE <−0.5 D (cycloplegic AR)	Smartphone	Prevalence	β = −0.09 (95% CI, −0.25 to −0.07)	Age, sex, season of app measurement, and operating system (iOS or Android)
Dong et al,^39^ 2022^a^^,^^b^	60.0	Cohort	14 296 Students (children, adolescents, and young adults)	Range, 7-18 y	China	SE <−0.5 D (VA, AR, and subjective refraction)	Online courses (smartphone, tablet, computer, and television)	Prevalence	<5 h/d, 1 [Reference]; ≥5 h/d, 1.40 (1.29 to 1.53)	Age, sex, province, provincial socioeconomic levels, and urban/rural areas
Zhang et al,^40^ 2022^a^	50.2	Cross-sectional	1401 adolescents and young adults	19.03 (2.78)	China	SE <−0.5 D in either eye (AR)	Digital device	Prevalence	Not often (0-2 h/d), 1 [Reference]; often (≥2 h/d), 1.406 (1.028 to 1.923)	Age, sex, types of sports, reading time, family history of myopia, education level, smoking, alcohol consumption, sleep deficiency, dietary bias, family income, BMI, and study locations
Mukazhanova et al,^41^ 2022^a^^,^^d^	28.3	Cross-sectional	2293 Secondary school–aged students (children and adolescents)	11.2 (3.6) years	Kazakhstan	SE <−0.5 D (cycloplegic AR)	Smartphone	Prevalence	None, 1 [Reference]; <1 h/d, 1.06 (0.64 to 1.75); 1-2 h/d, 1.23 (0.75 to 2.02); >2 h/d, 1.60 (0.95 to 2.67)	None
Wang et al,^42^ 2022^a^^,^^b^	10.7	Cross-sectional	23 930 Kindergarten-aged children	5.15 (0.37)	Taiwan	SE <−0.5 D (cycloplegic AR)	Smartphone, tablet, video game, computer, and television	Prevalence	<1 h/d on weekdays, 1 [Reference]; ≥1 h/d on weekdays, 1.20 (1.09 to 1.32)	Sex, duration of exposure to preventive strategies before eye examination, caregiver myopia, caregiver education, and time spent on after-school outdoor activities
Mohan et al,^43^ 2022^a^^,^^b^	Only patients with myopia included	Longitudinal	133 Children and adolescents	13.4 (3.29)	India	SE <−0.5 D (cyloplegic AR)	Smartphone use for video game	Progression	<1 h/d, 1 [Reference]; ≥1 h/d, 3.46 (NR)	Age, history of rapid progression prior to COVID-19, and sun exposure
Matsumura et al,^44^ 2022^a^^,^^d^	2.9	Cross-sectional	457 Children	4.77 (0.65)	Japan	SE <−0.5 D (VA and spot vision screener)	Smartphone, computer, or tablet	Prevalence	<1 h/d, 0 [Reference]; ≥1 h/d, β = 0.08 (95% CI, −0.05 to 0.20)	None
Makhdoum et al,^69^ 2023^a^^,^^b^	57.3	Cross-sectional	433 University students (young adults)	21.3 (2.0)	Saudi Arabia	Self-reported (questionnaire)	Digital device	Prevalence	None, 1 [Reference]; <1 h/d, 1.83 (0.05 to 63.41); 1-2 h/d, 1.12 (0.08 to 15.34); 2-3 h/d, 12.46 (1.67 to 92.94); >3 h/d, 5.47 (0.96 to 31.20)	History of having any eye disease, usual reading distance, frequency of performing visual assessment, and length of time spent outdoors every day
Cui et al,^45^ 2023^a^^,^^b^	57.2	Cohort	1496 Primary and secondary school–aged students	NR	China	SE <−0.5 D (VA and AR) or wearing orthokeratology lenses	Smartphone, tablet, computer, and television	Prevalence	<1 h/d, 1 [Reference]; 1-2.5 h/d, 1.021 (0.789-1.322); 2.5-4 h/d, 1.293 (0.700 to 2.391); ≥4 h/d, 0.790 (0.428 to 1.460)	Age, sex, urban and rural areas, city, poor eye habits, studying in a bright environment, light source, sleep quality, eye exercises, eating eggs, and drinking milk
Harrington,^46^ 2023^a^^,^^b^	3.7	Cross-sectional	723 School-aged children	7.08 (0.45)	Ireland	SE <−0.5 D (cycloplegic AR)	Smartphone, tablet, video game, computer, and television	Prevalence	>2h/d, 10.88 (4.35 to 27.24)	Age, sex, ethnicity, socioeconomic status, living environment, and parental myopia
Althnayan et al,^47^ 2023^a^	Only patients with myopia included	Longitudinal	150 Children and adolescents	11.0 (2.4)	Saudi Arabia	SE <−0.5 D in either eye (VA and cycloplegic refraction)	Digital device (phone, tablet, computer, and television)	Progression	Percentage of myopia progression: <2 h/d, 51%; 2-4 h/d, 68%; >4 h/d, 95%	None
Liu et al,^48^ 2023^a^	38.6	Cross-sectional	586 Children	Range, 6-12 y	China	SE <−0.5 D in either eye (cycloplegic AR)	Digital device (electronic devices and television)	Prevalence	Percentage of myopia prevalence: <7 h/wk, 35.1%; 7-14 h/wk, 40.2%; 14-21 h/wk, 46.0%; >21 h/wk, 58.3%	None
Pannu et al,^49^ 2023	50.0	Case-control	60 Children and adolescents	11.4 (Range, 5-15 y)	India	SE ≤−0.5 D (cycloplegic refraction)	Smartphone and computer	Prevalence	Percentage of myopia prevalence: <6 h/d, 36.7%; ≥6 h/d, 63.3%	None
Singh et al,^50^ 2023^a^^,^^b^	Only patients with myopia included	Longitudinal	200 Children, adolescents, or young adults	Range, 10-24 y	India	SE <−3.0 D in each eye (cycloplegic refraction)	Mobile phone and computer	Progression	Mobile phone: <2 h/d, 1 [Reference]; 2-4 h/d, 1.0 (0.4-1.9); 5-6 h/d, 1.2 (0.5-2.0); >6 h/d, 1.1 (0.6-1.9); Computer:<2 h/d, 1 [Reference]; 5-6 h/d, 0.3 (0.06-0.9); >6 h/d, 1.23 (1.1-1.7)	NR
Swetha,^51^ 2023^a^	45.0	Cross-sectional	100 Children	Range, 6-12 y	India	SE <−0.5 D (refraction)	Smartphone, tablet, and computer	Prevalence	Percentage of myopia prevalence: <2 h/d, 48.0%; 2-4 h/d, 42.5%; >4 h/d, 45.7%	Screen brightness, continuous screen usage, outdoor activity, nutritional status, and socioeconomic status
Zhang et al,^52^ 2023^b^	Only patients with myopia included	Cross-sectional	96 University students (adults)	Range, 18-28 y	China	SE <−0.5 D (AR)	Smartphone	Prevalence (of high myopia)	1.658 (1.264 to 2.246)	Vessel density of the inner retina at the macula, vessel density of radial peripapillary capillary at the optic disc, continuous near work time, and sleeping before or after midnight
Siska et al,^70^ 2023^a^^,^^b^	43.8	Cross-sectional	96 Students (adolescents)	16.5 (0.75)	Indonesia	Self-reported (questionnaire)	Gadget (tablet/laptop) or mobile phone	Prevalence	≤6 h/d, 1 [Reference]; >6 h/d, 9.733 (NR)	Light intensity, gadget usage position, distance, type of gadget, and genetic factor
Hu et al,^53^ 2024^a^^,^^b^	23.9	Cross-sectional	792 Students of grades 1-3 (children)	8.2 (1.6)	China	SE ≤−0.75 D (AR)	Digital devices	Prevalence	<2 h/d, 1 [Reference]; ≥2 h/d, 2.18 (1.18 to 4.00)	Age, maternal gestational hypertension, maternal education, height, paternal myopia, and maternal myopia
Huang et al,^55^ 2024^a^^,^^b^	71.3	Cross-sectional	126 375 Students (adolescents)	Range, 12-15 y	China	SE <−0.5 D (AR)	Smartphone and computer	Prevalence	<1 hr/d, 1 [Reference]; 1-3 h/d, 1.025 (0.996 to 1.055); >3 h/d, 1.061 (1.019 to 1.104)	Age, sex, place of residence, parents’ myopia condition, frequency of using eyes while lying down or leaning forward, frequency of using eyes while walking or riding in the car, frequency of outdoor exercise weekly, proper posture for reading and writing, distance from eyes to television screen, distance from eyes to computer screen, daily homework duration, and daily sleep duration
Kusumawardhany et al,^71^ 2024^a^^,^^d^	27.5	Cross-sectional	165 Adolescents	Range, 14-15 y	Indonesia	Self-reported (questionnaire)	Gadget or laptop	Prevalence	<6 h/d, 1 [Reference]; ≥6 h/d, 1.39 (1.04 to 1.85)	None
Zeng et al,^56^ 2024^a^^,^^f^	2-y incidence, 26%	Cohort	7006 Children	7.66 (1.18)	China	SE≤−0.5 D (AR)	Screen time	Incidence	<2 h/d, 1 [Reference]; ≥2 h/d, 3.080 (2.444 to 3.882)	Age, sex, spherical equivalent at baseline, school socioeconomic status, parental myopia, outdoor time, reading time, floor area ratio, and normalized difference vegetation index
Han et al,^57^ 2024^a^^,^^b^	60.2	Cross-sectional	3072 Adolescents and adults	Range, 15-59 y	Korea	SE <−0.75 D (AR)	Smartphone, tablet, and computer	Prevalence	<1 h/d, 1 [Reference]; 1-2 h/d, 1.35 (0.94 to 1.93); 3-4 h/d, 1.55 (1.08 to 2.23); >4 h/d, 1.75 (1.27 to 2.42)	Age, sex, education, residence area, occupation, smoking status, alcohol drinking frequency, reading (h/d), physical activities (h/d), sitting (h/d), hypertension, diabetes, and ophthalmologic examination
Gus et al,^58^ 2024^a^^,^^b^	17.4	Cross-sectional	330 Children	12.74 (NR)	Brazil	SE ≤−0.5 D (cycloplegic AR)	Time of electronic use	Prevalence	<4 h/d, 1 [Reference]; ≥4 h/d, 2.01 (1.31 to 3.09)	Age, sex, and skin color
Husein,^59^ 2024^a^^,^^d^	45.0	Cross-sectional	300 Children, adolescents, and adults	Range, 12-18	Indonesia	SE ≤−0.5 D (cycloplegic AR)	Electronic devices (eg, smartphones and computers)	Prevalence	<4 h/d, 1 [Reference]; ≥4 h/d, 2.54 (NR)	None
Zhao et al,^60^ 2024^a^^,^^b^	55.3	Cross-sectional	31 880 Children and adolescents	Mean range, 5.86-16.73 y across schools	China	SE ≤−0.5 D (VA and AR)	Computer game consoles, and television	Prevalence	Computer game consoles:<2 h/d, 1 [Reference]; ≥2 h/d, 0.928 (0.647 to 1.332); Television: <2 h/d, 1 [Reference]; ≥2 h/d, 1.323 (1.026 to 1.706)	Age, sex, and area

Abbreviations: AR, auto-refraction; BMI, body mass index; DASS-21, Depression, Anxiety and Stress Scale 21; D, diopters; NR, not reported; OR, odds ratio; PAQ-A, Physical Activity Questionnaire for Adolescents; SE, spherical equivalent; VA, visual acuity.

^a^ Studies with multiple levels of exposure that were included in the dose-response meta-analysis.

^b^ Multivariable analysis.

^c^ Because this study presented results by age group, it was included in the meta-analysis as 2 separate groups.

^d^ Univariate analysis.

^e^ Linear regression analyses of smartphone use (hr/d) during schooldays and SE.

^f^ Mixed-effect regression analysis.

### Linear Dose-Response Association

**Figure 1.**
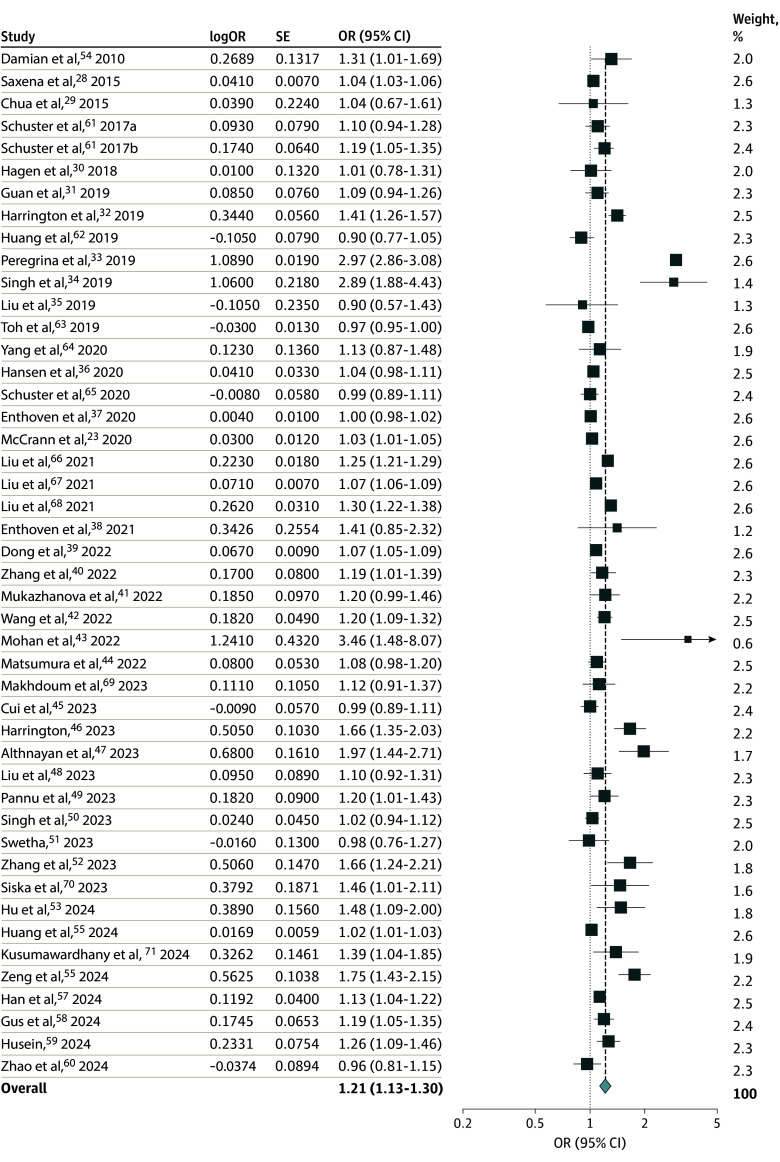
Risk Estimates for the Association of Additional Hour of Daily Digital Screen Time With Myopia The size of the box representing the point estimate for each study is in proportion to the contribution of that study’s weight estimate to the summary estimate. The horizontal lines indicate the 95% CIs. The diamond denotes the pooled odds ratio (OR), and the lateral tips of the diamond indicate the associated CIs. Because Shuster et al^61^ presented results by age group, it was included in the meta-analysis as 2 separate groups.

In the linear DRMA of the 45 studies, a daily 1-hour increment in digital screen time was associated with 21% higher odds of myopia (OR, 1.21; 95% CI, 1.13-1.30; *I*^2^, 99.0%) (Figure 1). When analyzed by specific outcomes, screen time consistently demonstrated an association with increased odds of myopia prevalence (OR, 1.19; 95% CI, 1.10-1.28) and progression (OR, 1.54; 95% CI, 1.01-2.36); results for myopia incidence were not statistically significant (OR, 1.40; 95% CI, 0.84-2.33) (eFigure 2 in Supplement 1). In the subgroup analysis stratified by study design, cross-sectional (OR, 1.21; 95% CI, 1.12-1.31) and cohort or longitudinal analyses (OR, 1.23; 95% CI, 1.03-1.47) yielded similar results (eFigure 3 in Supplement 1). In the subgroup analysis stratified by participant age, a significant association was observed across all age categories including 2 to 7 years (OR, 1.42; 95% CI, 1.12-1.78), 8 to 18 years (OR, 1.12; 95% CI, 1.07-1.18), and 19 years and older (OR, 1.16; 95% CI, 1.02-1.32) (eFigure 4 in Supplement 1). In addition, a subgroup analysis stratified by study country revealed significant associations in both Asian countries (OR, 1.17; 95% CI, 1.10-1.25) and countries outside of Asia (OR, 1.26; 95% CI, 1.06-1.51) (eFigure 5 in Supplement 1). According to the subgroup analysis stratified by whether screen devices were evaluated individually or in combination, the odds for myopia were significantly higher when devices were analyzed in combination (OR, 1.28; 95% CI, 1.15-1.42; *P* = .01) than for individual device analysis (OR, 1.09; 95% CI, 1.02-1.17) (eFigure 6 in Supplement 1). In the sensitivity analyses, the results aligned with those of the primary analysis (eFigures 7-10 in Supplement 1).

### Nonlinear Dose-Response Relationship

**Figure 2.**
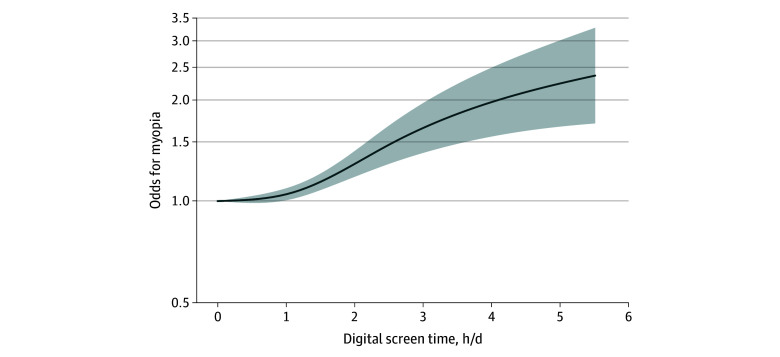
Dose-Response Curve for Additional Hour of Daily Digital Screen Time and Myopia The solid line represents risk estimates presented as odds ratios, and the shaded area represents the 95% CI.

A total of 34 studies^28,31,32,34,36,37,38,39,40,41,42,43,44,45,46,47,48,50,51,53,54,55,56,57,58,59,60,61,62,64,65,69,70,71^ (314 910 participants) with 104 dose groups were included in the nonlinear DRMA (Table 1 and eFigure 11 in Supplement 1). Table 2 displays the ORs for myopia across various levels of digital screen time exposure. Higher odds of myopia were associated with increasing screen time, ranging from 1 hour of daily exposure (OR, 1.05; 95% CI, 1.01-1.09) to 4 hours of daily exposure (OR, 1.97; 95% CI, 1.56-2.40). The dose-response curve indicates that the odds of myopia start to increase significantly with daily screen time of more than 1 hour (Figure 2). Beyond 4 hours per day, the rate of odds increase slowed, revealing a sigmoidal pattern. In the sensitivity analyses for nonlinear DRMA, the results were similar to those of the primary analysis (eFigure 12 in Supplement 1).

**Table 2.** Odds of Myopia Across Various Daily Digital Screen Time Exposures

Exposure levels of digital screen time, h/d	Myopia, OR (95% CI)
None	1 [Reference]
0.5	1.01 (0.99-1.04)
1.0	1.05 (1.01-1.09)
1.5	1.14 (1.08-1.21)
2.0	1.29 (1.18-1.41)
2.5	1.47 (1.29-1.68)
3.0	1.65 (1.39-1.96)
3.5	1.82 (1.48-2.24)
4.0	1.97 (1.56-2.40)
4.5	2.11 (1.62-2.76)
5.0	2.24 (1.67-3.01)

Abbreviation: OR, odds ratio.

### Publication Bias and Overall Quality of Evidence

Visual inspection of the funnel plot showed slight asymmetry, which could potentially indicate selective reporting. However, further statistical evaluation using an Egger test suggested no significant publication bias (intercept = 2.53; *t* = 1.63; *P* = .11) (eFigure 13 in Supplement 1). The overall certainty of evidence for the association of digital screen time with myopia at the outcome level was rated as low for both linear and nonlinear analyses (Table 3).

**Table 3.** Evaluation of Quality of Pooled Evidence Using the GRADE Framework^a^

Outcome	Participants, No.	Statistical heterogeneity	Risk of bias	Imprecision	Inconsistency	Indirectness	Publication bias	Quality of evidence (GRADE)
Linear dose-response association of digital screen exposure with myopia	335 524 (45 studies)	*I*^2^ = 99.0%	Not serious	Not serious	Serious	Not serious	Not serious	Low
Nonlinear dose-response association of digital screen exposure with myopia	314 910 (34 studies)	χ^2^_2_ = 29.9	Not serious	Not serious	Serious	Not serious	Not serious	Low

Abbreviation: GRADE, Grading of Recommendations, Assessment, Development, and Evaluation.

^a^ The quality of evidence for observational studies is graded starting at low quality for a causal effect and downgraded based on the following criteria: risk of bias,

imprecision, inconsistency, indirectness, and publication bias. The certainty rating was increased due to the observed dose-response gradient between digital screen exposure and myopia risk.

## Discussion

This systematic review and DRMA of 45 studies found that each additional hour of daily digital screen time was associated with significantly higher odds of myopia. The nonlinear DRMA demonstrated a sigmoidal pattern between digital screen time and myopia, with a pronounced increase in odds occurring between 1 and 4 hours of daily exposure. Notably, the association remained insignificant for screen time exposure of up to 1 hour per day, suggesting a potential safety threshold.

The association of prolonged near-vision work with increased risk of myopia has been well-established in numerous previous studies.^72,73,74,75,76^ The widespread adoption of smart devices among children introduces a novel dimension to our understanding and measurement of near-work activities. Global smartphone penetration surged from 21.6% in 2014 to 69.0% in 2023.^77^ Additionally, the age at which children begin using smart devices is decreasing, with many 2-year-olds spending up to 2 hours daily on such devices.^78^ As a quintessential form of near-vision work, the use of smart devices has been considered to have a significant association with increased risk of myopia.

Our findings differ from previous systematic reviews in several key aspects. Lanca et al^79^ analyzed 5 observational studies and found no significant association of screen time with myopia (OR, 1.02; 95% CI, 0.96-1.08). By contrast, a meta-analysis by Foreman et al,^12^ which included 11 observational studies, suggested that screen time on smart devices was associated with myopia (OR for smart devices alone, 1.26; 95% CI, 1.00-1.60; OR for combined smart device and computer use, 1.77, 95% CI, 1.28-2.45). Additionally, a recent meta-analysis^13^ found that screen time on computers (categorical OR, 8.19; 95% CI, 4.78-14.04) and televisions (categorical OR, 1.46; 95% CI, 1.02-2.10) was associated with myopia, whereas smartphone use was not. Our study demonstrated that when analyzing digital screen time comprehensively, including usage of smart devices such as smartphones, tablets, game consoles, computers, and televisions, there was not only a statistically significant association with myopia, but also evidence of a sigmoidal dose-response association as revealed through DRMA. This study offers an up-to-date and comprehensive analysis of the association of screen time with myopia, having incorporated detailed assessments of device types, study design, geographic regions, and participant age to uncover key patterns and influencing factors. By employing a DRMA, we further identified a potential safety threshold for screen time within a nonlinear framework, thus providing insights for public health and future research.

When interpreting the results of this analysis, it is important to note that we assessed the odds of myopia associated with screen time independently of other near-vision activities, such as reading or writing. It is also likely that digital screen use and other near-vision tasks collectively contribute to myopia risk, potentially influencing the overall dose-response trend. Therefore, caution is warranted when considering the 1-hour daily screen time safety threshold reported here. Another important consideration is that myopia was already prevalent in many Asian regions prior to the widespread use of digital devices^80^; this suggests that simply reducing screen time in favor of traditional near-vision activities may not be an effective prevention strategy. A more effective approach to the mitigation of myopia risk would involve minimizing overall near-work activities while promoting increased outdoor time.

In the subgroup analysis based on participants’ age, we observed a significant association across all age categories and found no statistically significant differences in ORs between age groups. However, given that factors such as myopia prevalence, progression rates, extent of other near-work activities, and cumulative exposure times to digital screens are likely to vary with participants’ age, differences in age across study populations may have contributed to the observed heterogeneity.

In our analysis, we identified significantly higher odds of myopia in studies examining combined digital device use compared with those examining single device use. When screen time is assessed by combining multiple devices rather than evaluating a single device, it is possible that total screen time is underreported, leading to a higher observed OR for myopia at equivalent screen time levels. Alternatively, interactions among different smart devices and their level of use could contribute to an increased myopia risk. However, it is crucial to interpret these findings cautiously because the included studies varied in the types of smart devices considered and lacked uniformity in outcome measurement methods and timing. This heterogeneity may lower the overall credibility of the proposed effect modification.

### Limitations

This study has limitations. First, some studies did not use objective measures to assess myopia. In addition, we did not analyze long-term fluctuations or temporal variations in digital screen time because most primary studies lacked repeated measurements. Future research using objective, serial assessments of digital screen time and myopia is needed to establish a more detailed dose-response pattern. Second, while most of the studies analyzed accounted for confounding factors associated with myopia risk, there was interstudy variability in how covariates were handled. Myopia is influenced by a series of risk factors, including a combination of genetic, environmental, and lifestyle factors, screen time being one of the latter, and potentially interacting with others. Therefore, the magnitude and pattern of the association of screen time with myopia may vary depending on which factors were adjusted for in individual studies. Third, the overall certainty of evidence at the outcome level was rated as low in our analysis. This downgrade was primarily due to inconsistent results from high heterogeneity, which indicates that the true effect may differ significantly from the estimated value. Fourth, the majority of the studies included in this analysis were cross-sectional, meaning that the associations reported cannot allow for derivation of causal relationships. It is essential to consider the possibility of confounding factors in the association of screen time with myopia. For instance, because screen use predominantly occurs indoors, the resulting reduction in exposure to the protective benefits of outdoor environments may contribute to the increased risk of myopia.

## Conclusions

This systematic review and DRMA found that digital screen time was associated with increased odds of myopia. The dose-response pattern showed a sigmoidal slope, indicating a potential safe range of 1 hour of daily screen exposure, with a notable rise in risk between 1 and 4 hours of exposure. These findings could offer meaningful insights for future research and inform educational strategies and public health policies aimed at addressing the myopia pandemic.

## Notes

Supplement 1. **eAppendix 1.** Search Term **eAppendix 2.** Newcastle-Ottawa Scale Assessment Scale **eMethods.** Methods for Converting Effect Sizes **eFigure 1.** Flow Diagram of Selection Process for Inclusion of Studies in Meta-Analysis **eAppendix 3.** References to Included Studies **eAppendix 4.** List of Articles Excluded at Full-Text Screening Stage\ **eTable 1.** Risk of Bias Assessment for Individual Studies **eFigure 2.** Subgroup Analysis Based on Myopia-Related Outcomes **eFigure 3.** Subgroup Analysis Based on Study Design **eFigure 4.** Subgroup Analysis Based on Participant Age **eFigure 5.** Subgroup Analysis Comparing Studies Conducted in Asia to Those Conducted Elsewhere **eFigure 6.** Subgroup Analysis Comparing Studies With Combined Device Analysis vs. Individual Device Analysis **eFigure 7.** Sensitivity Analysis Including Only Studies With Myopia Confirmed by Cycloplegic Refraction **eFigure 8.** Sensitivity Analysis Excluding Studies With Fewer Than 500 Participants **eFigure 9.** Sensitivity Analysis Excluding Studies With Unadjusted Data **eFigure 10.** Sensitivity Analysis Including Only Studies With Screen Time Exposure Limited to Smartphone Use **eFigure 11.** Association Between Digital Screen Time and Logarithm of Odds Ratio for Myopia Risk **eFigure 12.** Sensitivity Analyses of Dose-Outcome Curve **eFigure 13.** Contour-Enhanced Funnel Plots for the Primary Outcome (45 Studies)

Supplement 2.
Data Sharing Statement
