# Supplementary material for: Digital Screen Time and Myopia: A Systematic Review and Dose-Response Meta-Analysis
Source: JAMA Netw Open. 2025 Feb 21;8(2):e2460026. doi: 10.1001/jamanetworkopen.2024.60026 (PMC11846013; doi:10.1001/jamanetworkopen.2024.60026)

Supplemental Online Content

Ha A, Lee YJ, Lee M, Shim SR, Kim YK. Digital screen time and myopia in children, adolescents and adults: a systematic review and dose-response meta-analysis. *JAMA Netw. Open*. 2025;8(2):e2460026. doi:10.1001/jamanetworkopen.2024.60026

eAppendix 1. Search Term

eAppendix 2. Newcastle-Ottawa Scale Assessment Scale

eMethods. Methods for Converting Effect Sizes

eFigure 1. Flow Diagram of Selection Process for Inclusion of Studies in Meta-Analysis

eAppendix 3. References to Included Studies

eAppendix 4. List of Articles Excluded at Full-Text Screening Stage

eTable 1. Risk of Bias Assessment for Individual Studies

eFigure 2. Subgroup Analysis Based on Myopia-Related Outcomes

eFigure 3. Subgroup Analysis Based on Study Design

eFigure 4. Subgroup Analysis Based on Participant Age

eFigure 5. Subgroup Analysis Comparing Studies Conducted in Asia to Those Conducted Elsewhere

eFigure 6. Subgroup Analysis Comparing Studies With Combined Device Analysis vs. Individual Device Analysis

eFigure 7. Sensitivity Analysis Including Only Studies With Myopia Confirmed by Cycloplegic Refraction

eFigure 8. Sensitivity Analysis Excluding Studies With Fewer Than 500 Participants

eFigure 9. Sensitivity Analysis Excluding Studies With Unadjusted Data

eFigure 10. Sensitivity Analysis Including Only Studies With Screen Time Exposure Limited to Smartphone Use

eFigure 11. Association Between Digital Screen Time and Logarithm of Odds Ratio for Myopia Risk

eFigure 12. Sensitivity Analyses of Dose-Outcome Curve

eFigure 13. Contour-Enhanced Funnel Plots for the Primary Outcome (45 Studies)

This supplemental material has been provided by the authors to give readers additional information about their work.

© 2025 Ha A et al. *JAMA Network Open*.

Appendix 1. Search term

MEDLINE (Pubmed)

#1. Smartphone[Mesh] or Smartphone[Tiab]
#2. Cell Phone[Mesh] or "Cell Phone"[Tiab]
#3. Screen time[Mesh] or "Screen time"[Tiab]
#4. "Digital device"[Tiab]
#5. "Digital screen"[Tiab]
#6. Social Media[Mesh] or "Social Media"[Tiab]
#7. Video Games[Mesh] or "Video Games"[Tiab]
#8. Computers, Handheld[Mesh] or Computers[Tiab]
#9. Risk[Tiab] or "risk factor"[Tiab]
#10. #1 OR #2 OR #3 OR #4 OR #5 OR #6 OR #7 OR #8 OR #9
#11. Myopia[Mesh] or Myopia[Tiab]
#12. Myopic[Tiab]
#13. Refractive errors[Mesh] or "Refractive errors"[Tiab]
#14. Axial Length, Eye[Mesh] or "Axial Length"[Tiab]
#15. Refraction[Tiab]
#16. #11 OR #12 OR #13 OR #14 OR #15
#17. #10 AND #16

EMBASE (Ovid)

('Smartphone'/exp OR 'Mobile phone'/exp OR 'Screen time'/exp OR 'Social Media'/exp OR 'Video Game'/exp OR 'Computer'/exp OR 'Risk factor' OR 'Digital screen':ab,ti OR 'Digital device':ab,ti) AND ('Myopia'/exp OR 'Refraction error'/exp OR 'Eye axis length'/exp)

The Cochrane Library databases

#1 MeSH descriptor: [Smartphone] explode all trees
#2 MeSH descriptor: [Cell Phone] explode all trees
#3 MeSH descriptor: [Screen time] explode all trees
#4 MeSH descriptor: [Social Media] explode all trees
#5 MeSH descriptor: [Video Games] explode all trees
#6 MeSH descriptor: [Computers] explode all trees
#7 (Digital device): ti,ab,kw
#8 (Digital screen): ti,ab,kw
#9 Risk:ti,ab,kw
#10 (#1 OR #2 OR #3 OR #4 OR #5 OR #6 OR #7 OR #8 OR #9)
#11 MeSH descriptor: [Myopia] explode all trees
#12 MeSH descriptor: [Refractive errors] explode all trees
#13 MeSH descriptor: [Axial Length, Eye] explode all trees
#14 Myopic:ti,ab,kw
#15 Refraction:ti,ab,kw
#16 (#11 OR #12 OR #13 OR #14 OR #15)
#17 (#10 AND #16)

CINAHL

© 2025 Ha A et al. *JAMA Network Open.*

#1. 'Screen time' OR 'Smartphone' OR 'Cell Phones' OR 'Digital devices' OR 'Digital screens' OR 'Social Media' OR 'Video Games' OR 'Computer'
#2. 'Myopia' OR 'Refractive errors' OR 'Axial Length' OR 'Refraction'
#3. #1 AND #2
#4. PT Letter
#5. PT Editorial
#6. (MH Animals)
#7. #4 OR #5 OR #6
#8. #3 NOT #7
#9. Apply related words/Apply equivalent subjects/Exclude MEDLINE records

Clinicaltrials.gov

Condition/disease: 'Myopia' OR 'Refractive errors' OR 'Axial Length' OR 'Refraction'

Other terms: 'risk' OR 'risk factor' OR 'progression'

Intervention/treatment: 'Screen time' OR 'Smartphone' OR 'Cell Phones' OR 'Digital devices' OR 'Digital screens' OR 'Social Media' OR 'Video Games' OR 'Computer'

© 2025 Ha A et al. *JAMA Network Open*.

Appendix 2. Newcastle-Ottawa Scale assessment scale

For cross sectional studies

Selection: (Maximum 4 stars)

1) Representativeness of the sample:

a) Truly representative of the average in the target population (all subjects or random sampling). *

b) Somewhat representative of the average in the target population (non-random sampling). *

c) Selected group of users.

d) No description of the sampling strategy.

2) Sample size:

a) Justified and satisfactory (> 1000). *

b) Not justified.

3) Non-respondents:

a) Comparability between respondents and non-respondents characteristics is established, and the response rate is satisfactory. *

b) The response rate is unsatisfactory, or the comparability between respondents and non-respondents is unsatisfactory.

c) No description of the response rate or the characteristics of the responders and the non-responders.

4) Ascertainment of the exposure (risk factor):

a) Validated measurement tool. *

b) Non-validated measurement tool, but the tool is available or described. *

c) No description of the measurement tool.

Comparability: (Maximum 2 stars)

1) The subjects in different outcome groups are comparable, based on the study design or analysis. Confounding factors are controlled.

a) The study controls for the most important factor (age, and gender). *

b) The study control for any additional factor (outdoor activity/outdoor time). *

Outcome: (Maximum 3 stars)

1) Assessment of the outcome:

a) Independent blind assessment. **

b) Record linkage. *

c) Self report.

d) No description.

2) Statistical test:

a) The statistical test used to analyze the data is clearly described and appropriate, and the

© 2025 Ha A et al. *JAMA Network Open*.

measurement of the association is presented, including confidence intervals and the probability level (p value). *

b) The statistical test is not appropriate, not described or incomplete.

For cohort studies

Selection: (Maximum 4 stars)

1) Selection of the non exposed cohort:

a) truly representative of the average children and adolescents in the community. *

b) somewhat representative of the average children and adolescents who are less likely to use screen devices (describe) in the community. *

c) selected group of users eg nurses, volunteers.

d) no description of the derivation of the cohort.

2) Selection of the non exposed cohort:

a) drawn from the same community as the exposed cohort. *

b) drawn from a different source.

c) no description of the derivation of the non exposed cohort.

3) Ascertainment of exposure:

a) secure record (eg. Surgical records). *

b) structured interview. *

c) written self repot.

d) no description.

4) Demonstration that outcome of interest was not present at start of study:

a) yes. *

b) no.

Comparability: (Maximum 2 stars)

1) Comparability of cohorts on the basis of the design or analysis:

a) The study controls for the most important factor (age, and gender). *

b) The study control for any additional factor (outdoor activity/outdoor time). *

Outcome: (Maximum 3 stars)

1) Assessment of the outcome:

a) Independent blind assessment. *

b) Record linkage. *

c) Self report.

d) No description.

2) Was follow-up long enough for outcomes to occur:

a) yes (select an adequate follow up period for outcome of interest). *

b) no.

Adequacy of follow up of cohorts:

a) complete follow up - all subjects account for. *

b) subjects lost to follow up unlikely to introduce bias - small number lost - > 90 % follow up, or description provided of those lost). *

c) follow-up rate < 90 % and no description of those lost.

d) no statement

© 2025 Ha A et al. *JAMA Network Open*.

eMethods. Methods for converting effect sizes

$$\boldsymbol{Odds\ ratio = e^{b1}}$$

We applied the methodology used in the previous meta-analysis to convert regression coefficient to odds ratio.[1] When a logistic regression is calculated, the regression coefficient (b1) is the estimated increase in the log odds of the outcome per unit increase in the value of the exposure.[2]

References.

1. Foreman et al., Association between digital smart device use and myopia: a systematic review and meta-analysis. *Lancet Digit Health.* 2021;3(12):e806-e818.

2. Szumilas M. Explaining odds ratios. *J Can Acad Child Adolesc Psychiatry*. 2010;19(3):227.

© 2025 Ha A et al. *JAMA Network Open.*

eFigure 1. Flow diagram of selection process for inclusion of studies in meta-analysis

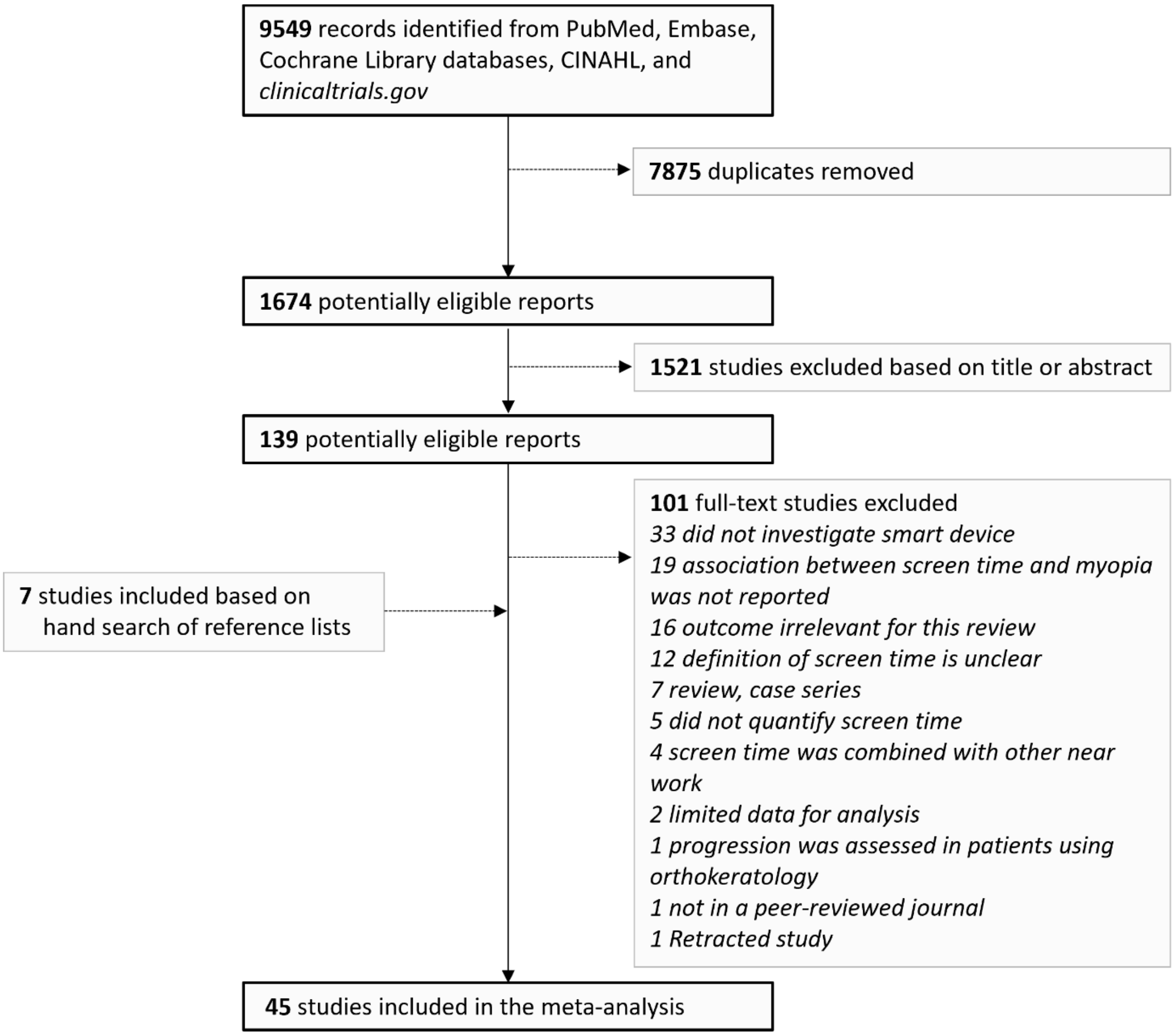

© 2025 Ha A et al. *JAMA Network Open*.

Appendix 3. References to included studies

1. Czepita D, Mojsa A, Ustianowska M, Czepita M, Lachowicz E. Reading, writing, working on a computer or watching television, and myopia. *Klinika Oczna/Acta Ophthalmol Polonica*. 2010;112(4):293-295.
2. Saxena R, Vashist P, Tandon R, et al. Prevalence of myopia and its risk factors in urban school children in Delhi: the North India Myopia Study (NIM Study). *PLoS One.* 2015;10(2):e0117349.
3. Chua SYL, Ikram MK, Tan CS, et al. Relative contribution of risk factors for early-onset myopia in young Asian children. *Invest Ophthalmol Vis Sci.* 2015;56(13):8101-8107.
4. Schuster AK, Elflein HM, Pokora R, Urschitz MS. Prevalence and risk factors of myopia in children and adolescents in Germany-results of the KiGGS survey. *Klinische Padiatrie.* 2017;229(4):234-240.
5. Hagen LA, Gjelle JV, Arnegard S, Pedersen HR, Gilson SJ, Baraas RC. Prevalence and possible factors of myopia in Norwegian adolescents. *Sci Rep.* 2018;8(1):13479.
6. Guan H, Yu NN, Wang H, et al. Impact of various types of near work and time spent outdoors at different times of day on visual acuity and refractive error among Chinese school-going children. *PLoS One.* 2019;14(4):e0215827.
7. Harrington SC, Stack J, O'Dwyer V. Risk factors associated with myopia in schoolchildren in Ireland. *Br J Ophthalmol.* 2019;103(12):1803-1809.
8. Huang L, Kawasaki H, Liu Y, Wang Z. The prevalence of myopia and the factors associated with it among university students in Nanjing: A cross-sectional study. *Medicine.* 2019;98(10):e14777.
9. Alvarez-Peregrina CC, Sanchez-Tena MA, Martinez-Perez CC, Villa-Collar CC. Prevalence and risk factors of myopia in Spain. *J Ophthalmol.* 2019;2019.
10. Singh NK, James RM, Yadav A, Kumar R, Asthana S, Labani S. Prevalence of myopia and associated risk factors in schoolchildren in North India. *Optom Vis Sci.* 2019;96(3):200-205.
11. Liu S, Ye S, Xi W, Zhang X. Electronic devices and myopic refraction among children aged 6-14 years in urban areas of Tianjin, China. *Ophthalmic Physiol Opt.* 2019;39(4):282-293.
12. Toh SH, Coenen P, Howie EK, Mukherjee S, Mackey DA, Straker LM. Mobile touch screen device use and associations with musculoskeletal symptoms and visual health in a nationally representative sample of Singaporean adolescents. *Ergonomics.* 2019;62(6):778-793.
13. Yang G-Y, Huang L-H, Schmid KL, et al. Associations between screen exposure in early life and myopia amongst Chinese preschoolers. *Int J Environ Res Public Health.* 2020;17(3):1056.
14. Hansen MH, Laigaard PP, Olsen EM, et al. Low physical activity and higher use of screen devices are associated with myopia at the age of 16-17 years in the CCC2000 Eye Study. *Acta Ophthalmol.* 2020;98(3):315-321.
15. Schuster AK, Krause L, Kuchenbäcker C, et al. Prevalence and time trends in myopia among children and adolescents: results of the German KiGGS study. *Dtsch Arztebl Int* 2020;117(50):855.
16. Enthoven CA, Tideman JWL, Polling JR, Yang-Huang J, Raat H, Klaver CC. The impact of computer use on myopia development in childhood: The Generation R study. *Prev Med.* 2020;132:105988.
17. McCrann S, Loughman J, Butler JS, Paudel N, Flitcroft DI. Smartphone use as a possible risk factor for myopia. *Clin Exp Optom.* 2021;104(1):35-41.
18. Liu J, Chen Q, Dang J. Examining risk factors related to digital learning and social isolation: youth visual acuity in COVID-19 pandemic. *J Glob Health.* 2021;11.
19. Liu J, Li B, Chen Q, Dang J. Student health implications of school closures during the COVID-19 pandemic: new evidence on the association of e-learning, outdoor exercise, and myopia. *Healthcare.* 2021 *9*(5): 500.
20. Liu J, Li B, Sun Y, Chen Q, Dang J. Adolescent vision health during the outbreak of COVID-19: association between digital screen use and myopia progression. *Front Pediatr.* 2021;9:662984.
21. Enthoven CA, Polling JR, Verzijden T, et al. Smartphone use associated with refractive error in teenagers: the myopia app study. *Ophthalmology.* 2021;128(12):1681-1688.
22. Dong Y, Jan C, Chen L, et al. The cumulative effect of multilevel factors on myopia prevalence, incidence, and progression among children and adolescents in China during the COVID-19 pandemic.

© 2025 Ha A et al. *JAMA Network Open.*

Transl Vis Sci Technol. 2022;11(12):9-9.
23. Zhang M, Sun Z, Zhu X, Zhang H, Zhu Y, Yan H. Sports and myopia: an investigation on the prevalence and risk factors of myopia in young sports-related groups in Tianjin, China. *Invest Ophthalmol Vis Sci.* 2022;63(6):27-27.
24. Mukazhanova A, Aldasheva N, Iskakbayeva J, et al. Prevalence of refractive errors and risk factors for myopia among schoolchildren of Almaty, Kazakhstan: a cross-sectional study. *PLoS One.* 2022;17(6):e0269474.
25. Wang C-Y, Hsu N-W, Yang Y-C, Chen Y-L, Shyong M-P, Tsai D-C. Premyopia at preschool age: population-based evidence of prevalence and risk factors from a serial survey in Taiwan. *Ophthalmology.* 2022;129(8):880-889.
26. Mohan A, Sen P, Peeush P, Shah C, Jain E. Impact of online classes and home confinement on myopia progression in children during COVID-19 pandemic: digital eye strain among kids (DESK) study 4. *Indian J Ophthalmol.* 2022;70(1):241-245.
27. Matsumura S, Dannoue K, Hori Y. Prevalence of myopia and its associated factors among Japanese preschool children. *Front Public Health.* 2022;10:901480.
28. Makhdoum H, Alrehaili A, Albelowi A, et al. Prevalence of Myopia and Its Related Factors Among University Students in Madinah, Saudi Arabia. *Cureus.* 2023;15(11).
29. Cui L, Zhou H, Lou X, et al. Effects of behaviors and surrounding environment on myopia before and during the COVID-19 pandemic: a longitudinal analysis of children and adolescents in China. *J Public Health.* 2023:1-13.
30. Harrington S, O'Dwyer V. The association between time spent on screens and reading with myopia, premyopia and ocular biometric and anthropometric measures in 6-to 7-year-old schoolchildren in Ireland. *Ophthalmic Physiol Opt.* 2023;43(3):505-516.
31. Althnayan YI, Almotairi NM, Alharbi MM, Alamer HB, Alqahtani HB, Alfreihi S. Myopia Progression Among School-Aged Children in the COVID-19 Distance-Learning Era. *Clin Ophthalmol.* 2023:283-290.
32. Liu Z, Wang Q, Zhao Q, et al. Association between whole-grain intake and myopia in chinese children: a cross-sectional epidemiological study. *BMC Ophthalmol.* 2023;23(1):1.
33. Pannu A, Vichare N, Pushkar K, Kumar A, Gupta S. Parallelism between hypovitaminosis D3 and recently detected myopia in children with amplified screen use in the COVID-19 era—A preliminary study. *Indian J Ophthalmol.* 2023;71(1):229-234.
34. Singh P, ChOudhary P. Impact of Electronic Gadgets Overuse on Myopia Progression among Young People: A Prospective Study. *J Clin Diagn Res*. 2023;17(2).
35. Swetha K. Evaluating the impact of digital screen use one paediatric myoipa development: A cross-sectional analysis. *Int J Acad Med Pharm*. 2023;5(6):243-246.
36. Zhang W, Hou X, Li C, et al. Influencing factors associated with high myopia in Chinese college students. *Front Med*. 2023;10:1146291.
37. Siska F, Siwi SW, Imam P, The relationship between gadget use and the incidenct of myopia among high school students during online learning. *Indonesian Journal of Community Health Nursing* 2023;8(2) 84-92.
38. Hu T, Wu R, Wang W, Li H, Peng X. Analysis of factors related to the development of ocular biometric parameters in Chinese children aged 6–10 years: a cross-sectional study. *BMJ Open.* 2024;14(2):e080066.
39. Huang Z, Song D, Tian Z, Wang Y, Tian K. Prevalence and associated factors of myopia among adolescents aged 12–15 in Shandong Province, China: a cross-sectional study. *Sci Rep*. 2024;14(1):17289.
40. Kusumawardhany R, Dharianta R. Factors related to the incidence of myopia in students at the junior high school 2 in mojokerto city. *Literasi Kesehatan Husada: Jurnal Informasi Ilmu Kesehatan.* 2024;8(2):45-50.
41. Zeng D, Yang Y, Tang Y, et al. Shaping school for childhood myopia: the association between floor area ratio of school environment and myopia in China. *Br J Ophthalmol.* 2024;

42. Han M, Jeong J, Yoon C, et al. Association between Near Work, Physical Activities and Myopia in Korean Adults During COVID-19 Outbreak. *Ophthalmic Epidemiol.* 2024:1-7.
43. Gus PI, Maman RSd, Lengler AD, et al. Prevalence of refractive errors under cycloplegia and associated factors with focus on the myopia epidemic among public school children from southern Brazil. *J Clin Exp Ophthalmol.* 2024;15(3):7
44. Husein M. Analysis of Risk Factors for Myopia in Adolescents in Urban Environments. *Int J Public Health*. 2024;1(3):159-172.
45. Zhao L, Jiang X, Zhang W, et al. Prevalence and risk factors of myopia among children and adolescents in Hangzhou. *Sci Rep.* 2024;14(1):24615.

© 2025 Ha A et al. *JAMA Network Open.*

Appendix 4. List of articles excluded at full-text screening stage

Author	Year	Study title	Reason for exclusion
Foster et al	2010	Refractive error, axial length and anterior chamber depth of the eye in British adults: the EPIC-Norfolk Eye Study.	Did not investigate smart devices
Borchert et al	2011	Risk factors for hyperopia and myopia in preschool children the multi-ethnic pediatric eye disease and Baltimore pediatric eye disease studies	Did not investigate smart devices
Gao et al	2011	Prevalence and characteristics of myopic retinopathy in a rural Chinese adult population: The Handan Eye Study.	Did not investigate myopia and smart devices
Chen et al	2012	Prevalence and associated risk factors of myopic maculopathy in elderly Chinese: The Shihpai eye study.	Did not investigate myopia and smart devices
Asakuma et al	2012	Prevalence and risk factors for myopic retinopathy in a japanese population: The hisayama study.	Did not investigate myopia (investigated myopic retinopathy instead) and smart devices
French et al	2013	Risk factors for incident myopia in Australian schoolchildren: The Sydney Adolescent Vascular and Eye Study	Did not investigate myopia and smart devices
Cheng et al	2013	Myopization factors affecting urban elementary school students in Taiwan	Did not investigate myopia and smart devices
Bastawrous et al	2013	Prevalence and predictors of refractive error and spectacle coverage in Nakuru, Kenya: a cross-sectional, population-based study	Did not investigate smart devices
Hsu et al	2016	Prevalence and risk factors for myopia in second-grade primary school children in Taipei: A population-based study	Digital screen time was categorized only as yes or no and was not quantified
Tsai et al	2016	Myopia Development Among Young Schoolchildren: The Myopia Investigation Study in Taipei	The association between screen time and myopia was not reported
Ansar et al	2016	Risk factors for refractive errors among school going childrens in Wah Cantonment, Rawalpindi, Pakistan.	Did not investigate smart devices
Czepita et al	2017	The Influence of Environmental Factors on the Prevalence of Myopia in Poland.	Screen time and near work activities, such as reading and writing, were analyzed together
Anamarina and Daniela-Edith	2017	Development and progression of refractive errors in medical students.	Irrelevant
Terasaki et al	2017	Association of lifestyle and body structure to ocular axial length in Japanese elementary school children	Only Spearman’s correlation analysis was reported
Hsu et al	2017	Risk factors for myopia progression in second-grade primary school children in Taipei: a population-based cohort study	Digital screen time was categorized only as yes or no and was not quantified
Ding et al	2018	Risk factors of myopia boom in young generation in China 'The Guangzhou Twin Eye Study	Did not specify near work

© 2025 Ha A et al. *JAMA Network Open*.

Ding et al	2018	Possible causes of discordance in refraction in monozygotic twins: Nearwork, time outdoors and stochastic variation.	Did not investigate smart devices
Bomotti et al	2018	Refraction and change in refraction over a 20-year period in the beaver dam eye study	Did not investigate smart devices
Adhikari et al	2018	Environmental factors associated with ocular morbidity among children in three ecological regions of Nepal: a phase II Nepal Pediatric Ocular Diseases Study	Myopia lumped together as refractive error and did not investigate smart devices
McCrann et al	2018	Parental attitudes to myopia: a key agent of change for myopia control?	The association between screen time and myopia was not reported
Bahhawi et al	2018	Refractive error among male primary school students in Jazan, Saudi Arabia: Prevalence and associated factors.	Did not investigate smart devices
Ahn et al	2018	The Influence of Parental Myopia on Children's Myopia in Different Generations of Parent-Offspring Pairs in South Korea.	Did not investigate smart devices
Cavazos-Salias et al	2019	Prevalence of myopia and associated risk factors in medical students in Monterrey	Did not investigate smart devices
Arafa et al	2019	Prevalence and risk factors of refractive errors among preparatory school students in Beni-Suef, Egypt	Did not investigate smart devices
Flitcroft et al	2019	The spatial frequency content of urban and indoor environments as a potential risk factor for myopia development	Did not investigate smart devices
Luong et al	2020	Racial and Ethnic Differences in Myopia Progression in a Large, Diverse Cohort of Pediatric Patients	Investigated the analytical model best describing myopia progression
Do et al	2020	Association between Time Spent on Smart Devices and Change in Refractive Error: A 1-Year Prospective Observational Study among Hong Kong Children and Adolescents	Only the differences in spherical equivalent change were reported.
Abdiyeva	2020	Dependence of the progression rate of myopia among children from the place of residence	Did not investigate smart devices
Alomair et al	2020	The prevalence and predictors of refractive error among school children in Riyadh, Saudi Arabia	Digital screen time was categorized only as yes or no and was not quantified
Brennan et al	2020	Annual myopia progression and subsequent year progression in Singaporean children	Did not investigate smart devices
Cao	2020	Epidemiology of refractive and associated factors in school-aged children in Xiangtan	Did not investigate smart devices
Hung et al	2020	The prevalence of myopia and factors associated with it among secondary school children in rural Vietnam	Did not investigate smart devices
Liu et al	2020	Sleeping late is a risk factor for myopia development amongst school-aged children in China	Did not investigate smart devices
Shuker et al	2020	The biophysical efficacy of smart phones on the eyes of children from 3-12 years	The association between screen time and myopia was not reported
Wang	2020	Prevalence and influencing factors of myopia in adolescents and children in Gaoping, Shanxi Province	Did not investigate smart devices
Zhang et al	2020	Effect of Genetic-Environmental Interaction on Chinese Childhood Myopia	Did not investigate smart devices
Gupta et al	2021	Outdoor activity and myopia progression in children: A follow-up study using mixed-effects	Did not investigate smart devices

© 2025 Ha A et al. *JAMA Network Open*.

		model	
Hu et al	2021	Rates of Myopia Development in Young Chinese Schoolchildren During the Outbreak of COVID-19	Did not investigate smart devices
Lanca et al	2021	Rapid myopic progression in early childhood is associated with teenage high myopia	Did not investigate smart devices
Li et al	2021	Effect of School-Based Family Health Education via Social Media on Children's Myopia and Parents' Awareness: A Randomized Clinical Trial	The association between screen time and myopia was not reported
Liu et al	2021	Relationship between myopia progression and school entrance age: A 2.5-year longitudinal study	Did not investigate smart devices
Lu et al	2021	Association of Myopia in Elementary School Students in Jiaojiang District, Taizhou City, China	Digital screen time was categorized only as yes or no and was not quantified
Öztürk et al	2021	The effects of smartphone, tablet and computer overuse on children's eyes during the COVID-19 pandemic	Observed changes over time within a single group
Rahman et al	2021	Determinants of early refractive error on school-going children (10-12 years) in Dhaka City, Bangladesh	Did not investigate smart devices
Shrestha et al	2021	Prevalence of Refractive Error and Ocular Pathologies among School Children: Finding from the School Eye Program of Dhulikhel Hospital	Did not investigate smart devices
Yang et al	2021	The associations of high academic performance with childhood ametropia prevalence and myopia development in China	Definition of screen time is unclear
Malik et al	2022	Prevalence and Risk Factors of Myopia among Medical students	The association between screen time and myopia was not reported
Cai et al	2022	Complex Interplay Between COVID-19 Lockdown and Myopic Progression	Observed changes over time within a single group
Kiruthi et al	2022	Refractive Errors Due To Online Classes Among School Children During The Covid-19 Pandemic	The association between screen time and myopia was not reported
Liu et al	2022	Refractive Errors and Risk Factors for Myopia in Primary School Students in Urumqi	Retracted study
Babu et al	2022	A study to assess the impact of increased screen time during COVID-19 pandemic on eye health	Did not investigate myopia
Ma et al	2022	Effect of Parental Myopia on Change in Refraction in Shanghai Preschoolers: A 1-Year Prospective Study	Did not investigate smart devices
Pardhan et al	2022	Risks of Digital Screen Time and Recommendations for Mitigating Adverse Outcomes in Children and Adolescents	Narrative review
Servey et al	2022	Clinical Profile of Refractive Errors Associated with Screen Time in Children Aged 5-15 Years in a Tertiary Care Hospital of Southern India during COVID Pandemic: A Prospective Study	The association between screen time and myopia was not reported
AlShamlan et al	2023	Myopia progression in school children with prolonged screen time during the coronavirus disease confinement	The association between screen time and myopia was not reported
Chen et al	2023	Axial Length Elongation Profiles from 3 to 6 years old in an Asian Paediatric Population: the Growing Up in Singapore Towards health Outcomes birth cohort study (GUSTO)	Observed changes over time within a single group
Shovna et al	2023	Progression in refractive error in children during COVID-19 pandemic due to virtual classes: A	The association between screen time and myopia was

		cohort study	not reported
Kim and Choi	2023	Association between dietary nutrient intake and prevalence of myopia in Korean adolescents: evidence from the 7th Korea National Health and Nutrition Examination Survey	Did not investigate smart devices
Lone et al	2023	Determinants of Refractive Errors on School-going Children Attending Ophthalmic Clinic of AlMoosa Hospital in Saudi Arabia	Definition of screen time is unclear
Nie et al	2023	Epidemiological study of refractive errors in children and adolescents of Han and Li ethnics in the Ledong and Wanning areas of Hainan Province	Did not investigate smart devices
Singh et al	2023	Competitive Environment and Myopia Progression in Young People: A Prospective Study	Did not investigate smart devices
Worku et al	2023	The Magnitude of Refractive Error and Its Associated Factors Among Patients Visiting Ophthalmology Clinics in Southern Ethiopia, 2022	Did not quantify screen time
Zhang et al	2023	Clouclip combined with a questionnaire on the influence factors of myopia in children	Did not investigate smart devices
Zhu et al	2023	Myopia among children and adolescents: an epidemiological study in Fuzhou City	Did not investigate risk factors for myopia
Singh et al	2023	The Association between Digital Screen Time and Myopia among Children of Age Group 5 Year to 15 Year Presenting at a Tertiary Care Medical Centre	The association between screen time and myopia was not reported
Zhang et al	2023	Prevalence of Myopia in Children Before, During, and After COVID-19 Restrictions in Hong Kong	The association between screen time and myopia was not reported
Chang et al	2023	Orthokeratology compliance, digital device use, and myopia control among children with myopia during COVID-19 home confinement in Taiwan.	Myopia progression was assessed in patients using orthokeratology
Güemes-Villahoz et al	2024	Lifestyle Factors in Myopic Spanish Children	Definition of screen time is unclear
Yang et al	2024	The prevalence of myopia remains stable under tighter COVID-19 social restriction in preschoolers receiving a school-based eyecare program	The association between screen time and myopia was not reported
Zong et al	2024	Effects of outdoor activity time, screen time, and family socioeconomic status on physical health of preschool children	The association between screen time and myopia was not reported
Yuan et al	2024	The gap between parental knowledge and children practice of myopia control and challenge under COVID-19: a web-based survey in China	Did not investigate smart devices
Yang et al	2024	The prevalence of myopia remains stable under tighter COVID-19 social restriction in preschoolers receiving a school-based eyecare program	Observed changes over time within a single group
Bikbov et al	2024	Prevalence and associated factors of myopia in children and adolescents in Russia: the Ural Children Eye Study	Screen time was combined with book reading time for analysis
Shafie	2024	Exploring the Influence of Screen Time on Myopia Risk in Preschoolers and Adults	Narrative review
Olawale et al	2024	Assessing The Relationship Between Screen Time and Myopia In Children Aged 5-12 Ibadan South-West Local Government	Definition of screen time is unclear

© 2025 Ha A et al. *JAMA Network Open*.

Ruoyi et al	2024	Correlation analysis of electronic screen use and myopia among primary and secondary school students in six provinces and cities of China	Definition of screen time is unclear
Alah et al	2024	Screen Time Soars and Vision Suffers: How School Closures During the Pandemic Affected Children and Adolescents' Eyesight	The association between screen time and myopia was not reported
Anshari et al	2024	Myopia Progression in Children: A Comparative Analysis of Prepandemic and pandemic periods	The association between screen time and myopia was not reported
Ba et al	2024	The impact of lifestyle factors on myopia development: Insights and recommendations	Narrative review
Sano et al	2024	Association between ocular diseases and screen time and sedentary time derived from job-exposure matrices	The association between screen time and myopia was not reported
Biswas et al	2024	The influence of the environment and lifestyle on myopia	Narrative review
Sahoo et al	2024	Vision problems and screen time exposure among adolescents in rural schools: a cross-sectional study"	The association between screen time and myopia was not reported
Lacsa	2024	Evaluating the multifaceted approach to myopia prevention: beyond outdoor time for overweight children	Letter to the editor
Ljubic et al	2024	Risk Factors in Adult Myopia in Macedonia	Screen time was combined with book reading time for analysis
Guo et al	2024	Associations of incident myopia and its development with related factors among school-aged children across different grades: a four-year longitudinal study	A longitudinal study that estimated changes in non-myopic children
Luo et al	2024	Analysis of the prevalence and related factors for comorbidity of myopia, obesity, and depression symptoms among middle school students in Beijing	Definition of screen time is unclear
Li et al	2024	Lifestyle and risk of developing myopia in school children in Chongqing, China	Definition of screen time is unclear
Zeng et al	2024	Myopia Progression in School-Age Children During the COVID-19 Pandemic	Definition of screen time is unclear
Özısık et al	2024	Myopia Progression in School-Age Children During the COVID-19 Pandemic	Did not investigate smart devices
He et al	2024	Survey on pattern of myopia in school children in Hangzhou after the COVID-19 pandemic: a school-based vision screening study	Did not investigate smart devices
Borelli et al	2024	Prevalence of myopia, outdoor exposure and screen time at university entrance in Bahía Blanca urban environment	Definition of screen time is unclear
Salman	2024	Electronic Device Use in College Students and Impact of Screen Setting Choices on Choroidal Thickness and Myopia Progression Risk	Not published in a peer-reviewed journal
Almutairi et al	2024	Prevalence and Interrelationships of Screen Time, Visual Disorders, and Neck Pain Among University Students: A Cross-Sectional Study at Majmaah University	The association between screen time and myopia was not reported
Wang et al	2024	Prevalence of myopia and refractive parameters among children and adolescents in Hi-tech District of Chengdu City (2021-2023)	The association between screen time and myopia was not reported
Deng et al	2024	Analysis of risk factors associated with pre-myopia among primary school students in the Mianyang Science City	Analyzed risk factors for pre-myopia rather than myopia

© 2025 Ha A et al. *JAMA Network Open*.

Huo et al	2024	Myopia: Its Status and Environmental Correlations Among School Students in Fushun, China	Definition of screen time is unclear
Liu et al	2024	The Prevalence and Risk Factors of Abnormal Vision Among Preschool Children	The diagnostic criteria for myopia are unclear
Martelli et al	2024	Prevalence of myopia and associated risk factors in children and adolescents at an eye hospital in western Paraná	Did not investigate smart devices
Li et al	2024	Influence of eye-related behavior on myopia among junior middle school students under the background of double reduction during the COVID-19 pandemic	Screen time was combined with book reading time for analysis
Sarkar et al	2024	A Study on Prevalence and Risk Factors of Refractive Errors among Undergraduate Medical Students in a Tertiary Care Hospital of West Bengal	Definition of screen time is unclear
Kurniawan et al	2024	Descriptive on The Factors of Myopia on Medical Students of Muslim University of Indonesia	Definition of screen time is unclear

© 2025 Ha A et al. *JAMA Network Open*.

eTable 1. Risk of bias assessment for individual studies

Risk of bias assessment for individual studies (cross-sectional studies)

Study	Selection				Comparability	Outcome		Total score
	Representa ctiveness of the sample	Sample size	Non-respondent s	Ascertainme nt of exposure	Based on design and analysis	Assessment of outcome	Statistical test	
Damian et al, 2010	★	★		★		★★		5
Saxena et al, 2015	★	★		★	★★	★★	★	8
Schuster et al, 2017	★	★		★	★★		★	6
Hagen et al, 2018	★		★	★	★	★★	★	7
Guan et al, 2019	★	★	★	★	★★	★★	★	9
Harrington et al, 2019	★	★		★	★★	★★	★	8
Huang et al, 2019	★	★	★	★			★	5
Alvarez-Peregrina et al, 2	★	★	★	★	★	★★		7
Singh et al, 2019	★	★		★	★★	★★	★	8
Liu et al, 2019	★			★	★★	★★	★	7
Toh et al, 2019	★	★		★	★★		★	6
Yang et al, 2020	★	★		★	★★		★	6
Schuster et al, 2020	★	★		★	★★		★	6
McCrann et al, 2020	★		★	★	★★		★	6
Liu et al, 2021a	★	★		★	★★		★	6
Liu et al, 2021b	★	★		★	★★		★	6
Liu et al, 2021	★	★		★	★★		★	6
Zhang et al, 2022	★	★		★	★★	★★	★	8
Mukazhanova et al, 2022	★	★	★	★		★★	★	7
Wang et al, 2022	★	★		★	★★	★★	★	8
Matsumura et al, 2022	★		★	★	★★	★★	★	8
Makhdoum et al, 2023	★			★	★★		★	5
Harrington, 2023	★			★	★★	★★	★	7
Liu et al, 2023	★		★	★	★	★★	★	7
Pannu et al, 2023	★		★	★		★★	★	6
Swetha, 2023	★			★	★★	★★	★	7
Zhang et al, 2023	★		★	★	★★	★★	★	8
Hu et al, 2024	★		★	★	★★	★★	★	8
Siska et al, 2023	★		★	★	★★			5
Huang et al, 2024	★	★		★	★★	★★	★	8
Kusumawardhany et al, 2	★		★	★				3
Han et al, 2024	★	★	★	★	★★	★★	★	9
Gus et al, 2024	★		★	★	★	★★	★	7
Husein, 2024	★			★		★★	★	5
Zhao et al, 2024	★	★	★	★	★	★★	★	8

© 2025 Ha A et al. *JAMA Network Open*.

Risk of bias assessment for individual studies (cohort studies)

Study	Selection				Comparability	Outcome		Total score
	Representativeness of the exposed cohort	Selection of the non exposed cohort	Ascertainment of exposure	Outcome was not present at start of study	Based on design and analysis	Assessment	Follow-up	
Chua et al, 2015	★	★		★	★★	★	★	7
Hansen et al, 2020	★	★		★	★★	★	★	7
Enthoven et al, 2020	★	★		★	★	★	★★	7
Enthoven et al, 2021	★	★	★	★	★★	★	★	8
Dong et al, 2022	★	★		★	★★	★	★	7
Mohan et al, 2022	★	★			★★	★	★★	7
Cui et al, 2023	★	★		★	★★	★	★★	8
Althnayan et al, 2023	★	★		★	★	★	★★	7
Singh and Choudhary	★	★		★	★	★	★★	7
Zeng et al, 2024	★	★		★	★★	★	★★	8

For each of the sections Selection, Comparability, and Outcome/Exposure, a maximum of 4, 2, and 3 points could be given, respectively. Thus the maximum score was 9. A higher score means higher quality.

© 2025 Ha A et al. *JAMA Network Open*.

eFigure 2. Subgroup analysis based on myopia-related outcomes. OR = odds ratio; CI = confidence interval. g8=1 for incidence; g8=2 for prevalence; g8=3 for progression.

Study	logOR	SE(logOR)	OR	95%-CI	Weight
**g8 = 2**					
Damian et al, 2010	0.2689	0.1317	1.31	[1.01; 1.69]	2.0%
Saxena et al, 2015	0.0410	0.0070	1.04	[1.03; 1.06]	2.6%
Schuster et al, 2017a	0.0930	0.0790	1.10	[0.94; 1.28]	2.3%
Schuster et al, 2017b	0.1740	0.0640	1.19	[1.05; 1.35]	2.4%
Hagen et al, 2018	0.0100	0.1320	1.01	[0.78; 1.31]	2.0%
Guan et al, 2019	0.0850	0.0760	1.09	[0.94; 1.26]	2.3%
Harrington et al, 2019	0.3440	0.0560	1.41	[1.26; 1.57]	2.5%
Huang et al, 2019	-0.1050	0.0790	0.90	[0.77; 1.05]	2.3%
Peregrina et al, 2019	1.0890	0.0190	2.97	[2.86; 3.08]	2.6%
Singh et al, 2019	1.0600	0.2180	2.89	[1.88; 4.43]	1.4%
Liu et al, 2019	-0.1050	0.2350	0.90	[0.57; 1.43]	1.3%
Toh et al, 2019	-0.0300	0.0130	0.97	[0.95; 1.00]	2.6%
Yang et al, 2020	0.1230	0.1360	1.13	[0.87; 1.48]	1.9%
Hansen et al, 2020	0.0410	0.0330	1.04	[0.98; 1.11]	2.5%
Schuster et al, 2020	-0.0080	0.0580	0.99	[0.89; 1.11]	2.4%
Enthoven et al, 2020	0.0040	0.0100	1.00	[0.98; 1.02]	2.6%
McCrann et al, 2020	0.0300	0.0120	1.03	[1.01; 1.05]	2.6%
Liu et al, 2021a	0.2230	0.0180	1.25	[1.21; 1.29]	2.6%
Liu et al, 2021b	0.0710	0.0070	1.07	[1.06; 1.09]	2.6%
Enthoven et al, 2021	0.3426	0.2554	1.41	[0.85; 2.32]	1.2%
Dong et al, 2022	0.0670	0.0090	1.07	[1.05; 1.09]	2.6%
Zhang et al, 2022	0.1700	0.0800	1.19	[1.01; 1.39]	2.3%
Mukazhanova et al, 2022	0.1850	0.0970	1.20	[0.99; 1.46]	2.2%
Wang et al, 2022	0.1820	0.0490	1.20	[1.09; 1.32]	2.5%
Matsumura et al, 2022	0.0800	0.0530	1.08	[0.98; 1.20]	2.5%
Makhdoum et al, 2023	0.1110	0.1050	1.12	[0.91; 1.37]	2.2%
Cui et al, 2023	-0.0090	0.0570	0.99	[0.89; 1.11]	2.4%
Harrington, 2023	0.5050	0.1030	1.66	[1.35; 2.03]	2.2%
Liu et al, 2023	0.0950	0.0890	1.10	[0.92; 1.31]	2.3%
Pannu et al, 2023	0.1820	0.0900	1.20	[1.01; 1.43]	2.3%
Swetha, 2023	-0.0160	0.1300	0.98	[0.76; 1.27]	2.0%
Zhang et al, 2023	0.5060	0.1470	1.66	[1.24; 2.21]	1.8%
Siska et al, 2023	0.3792	0.1871	1.46	[1.01; 2.11]	1.6%
Hu et al, 2024	0.3890	0.1560	1.48	[1.09; 2.00]	1.8%
Huang et al, 2024	0.0169	0.0059	1.02	[1.01; 1.03]	2.6%
Kusumawardhany et al, 2024	0.3262	0.1461	1.39	[1.04; 1.85]	1.9%
Han et al, 2024	0.1192	0.0400	1.13	[1.04; 1.22]	2.5%
Gus et al, 2024	0.1745	0.0653	1.19	[1.05; 1.35]	2.4%
Husein, 2024	0.2331	0.0754	1.26	[1.09; 1.46]	2.3%
Zhao et al, 2024	-0.0374	0.0894	0.96	[0.81; 1.15]	2.3%
**Random effects model**			**1.19**	**[1.10; 1.28]**	**89.1%**
Heterogeneity: I^2 = 99%, p = 0					
**g8 = 1**					
Chua et al, 2015	0.0390	0.2240	1.04	[0.67; 1.61]	1.3%
Zeng et al, 2024	0.5625	0.1038	1.75	[1.43; 2.15]	2.2%
**Random effects model**			**1.40**	**[0.84; 2.33]**	**3.5%**
Heterogeneity: I^2 = 78%, p = 0.03					
**g8 = 3**					
Liu et al, 2021c	0.2620	0.0310	1.30	[1.22; 1.38]	2.6%
Mohan et al, 2022	1.2410	0.4320	3.46	[1.48; 8.07]	0.6%
Althnayan et al, 2023	0.6800	0.1610	1.97	[1.44; 2.71]	1.7%
Singh and Choudhary, 2023	0.0240	0.0450	1.02	[0.94; 1.12]	2.5%
**Random effects model**			**1.54**	**[1.01; 2.36]**	**7.4%**
Heterogeneity: I^2 = 91%, p < 0.01					
**Random effects model**			**1.21**	**[1.13; 1.30]**	**100.0%**

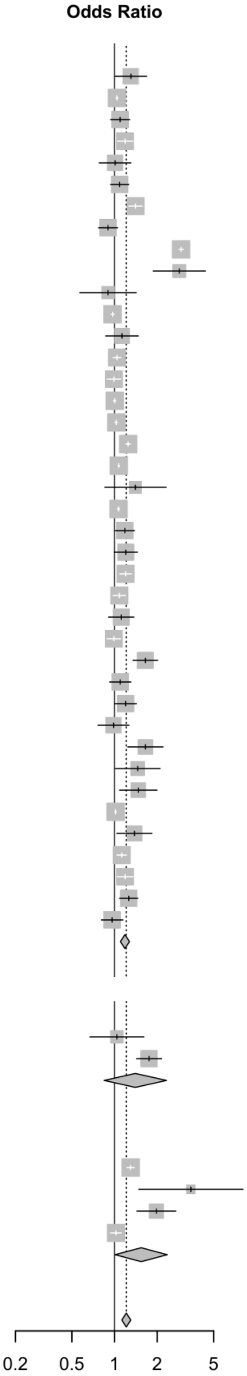

Heterogeneity: I^2 = 99%, p = 0
Test for subgroup differences: χ^2_2 = 1.80, df = 2 (p = 0.41)

© 2025 Ha A et al. *JAMA Network Open*.

eFigure 3. Subgroup analysis based on study design. OR = odds ratio; CI = confidence interval. g1=1 for cross-sectional study; g1=2 for cohort or longitudinal study.

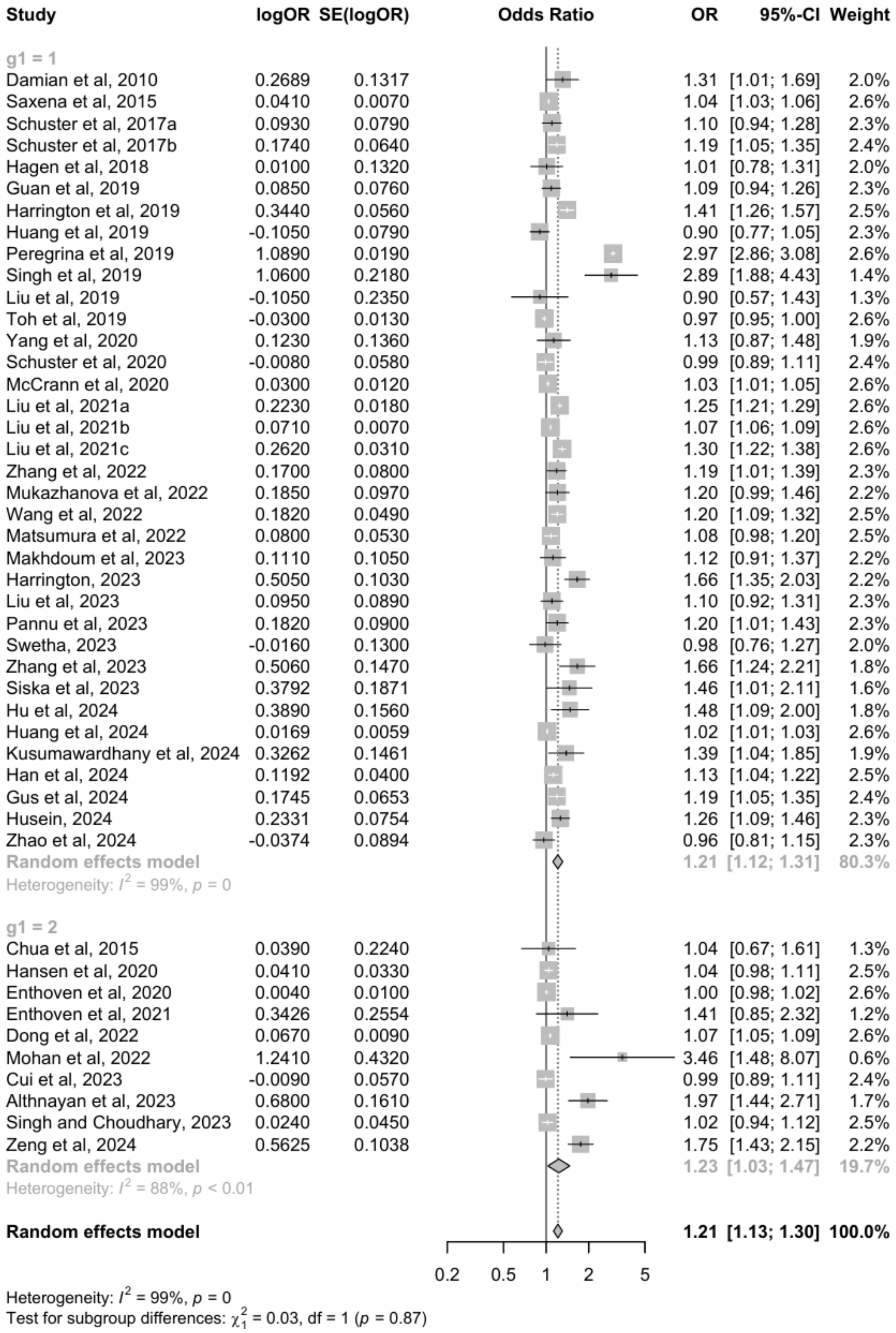

© 2025 Ha A et al. *JAMA Network Open*.

eFigure 4. Subgroup analysis based on participant age. OR = odds ratio; CI = confidence interval. g3=1 for 2-7 years; g3=2 for 8-18 years; g3=3 for studies including participants aged 19 years and older.

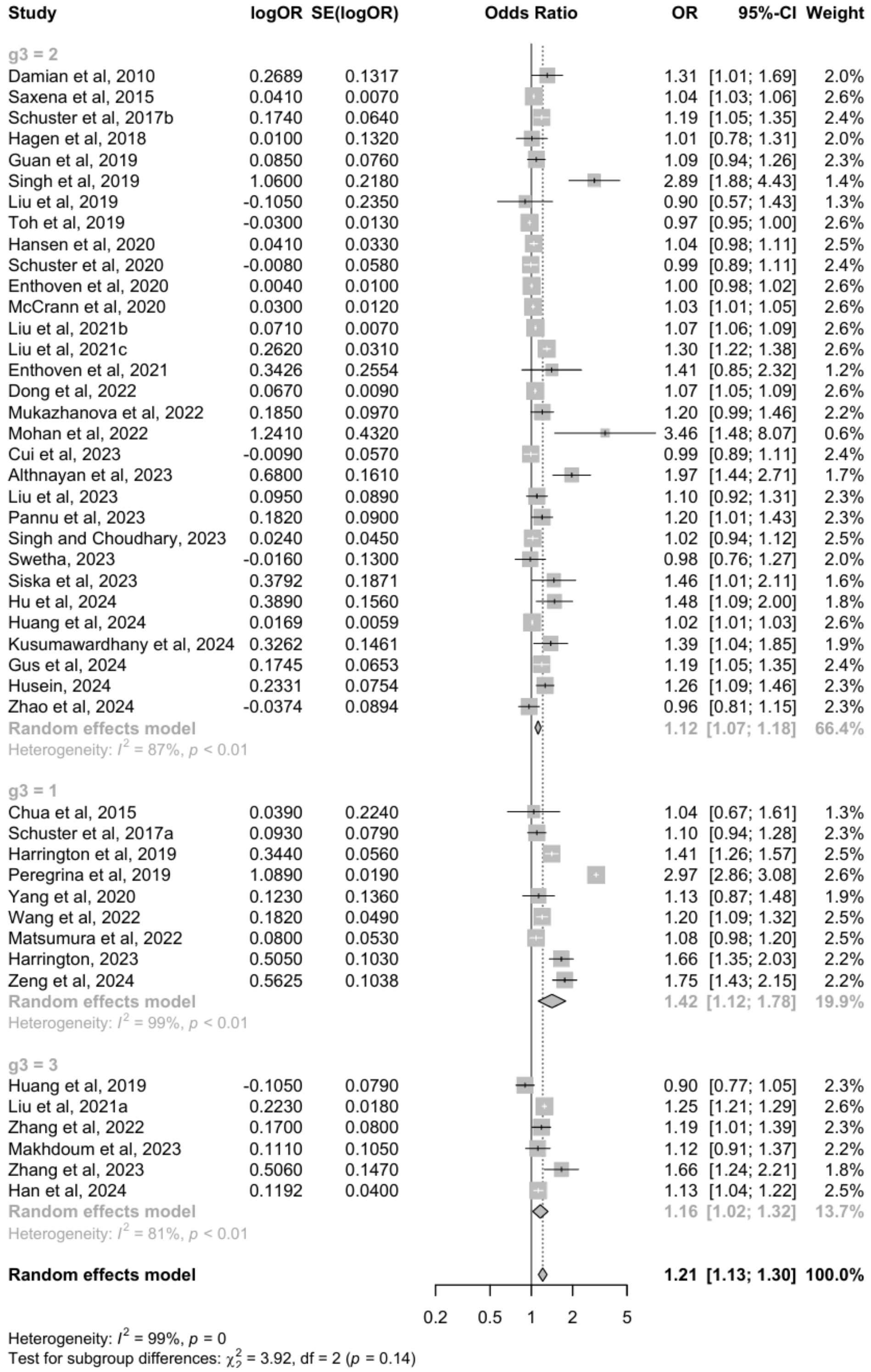

eFigure 5. Subgroup analysis comparing studies conducted in Asia to those conducted elsewhere. OR = odds ratio; CI = confidence interval. g4=1 for Asia; g4=2 for non-Asia regions.

© 2025 Ha A et al. *JAMA Network Open*.

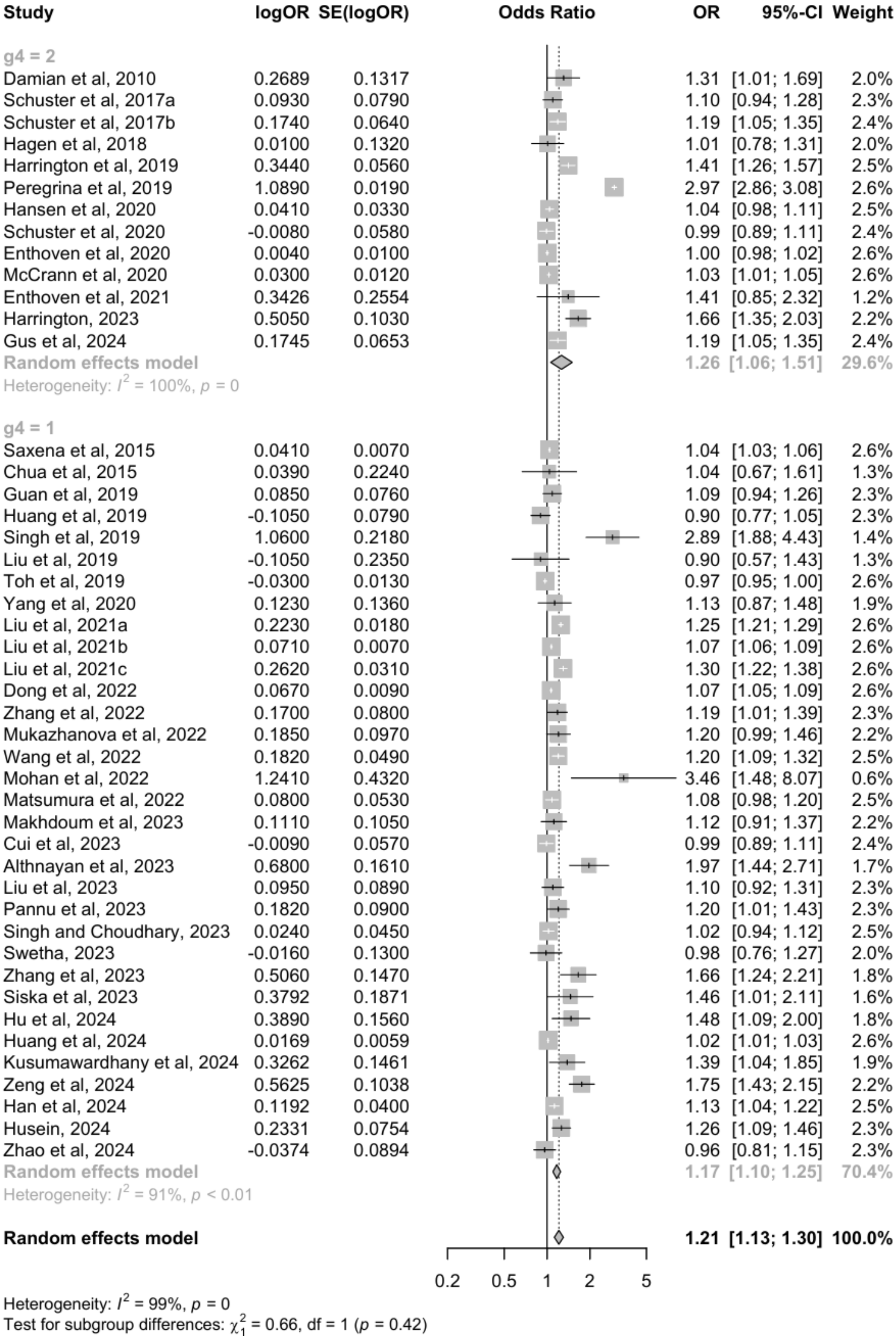

eFigure 6. Subgroup analysis comparing studies with combined device analysis vs. individual device analysis. OR = odds ratio; CI = confidence interval. g6=1 for single digital device analysis; g6=2 for combined digital device analysis.

© 2025 Ha A et al. *JAMA Network Open*.

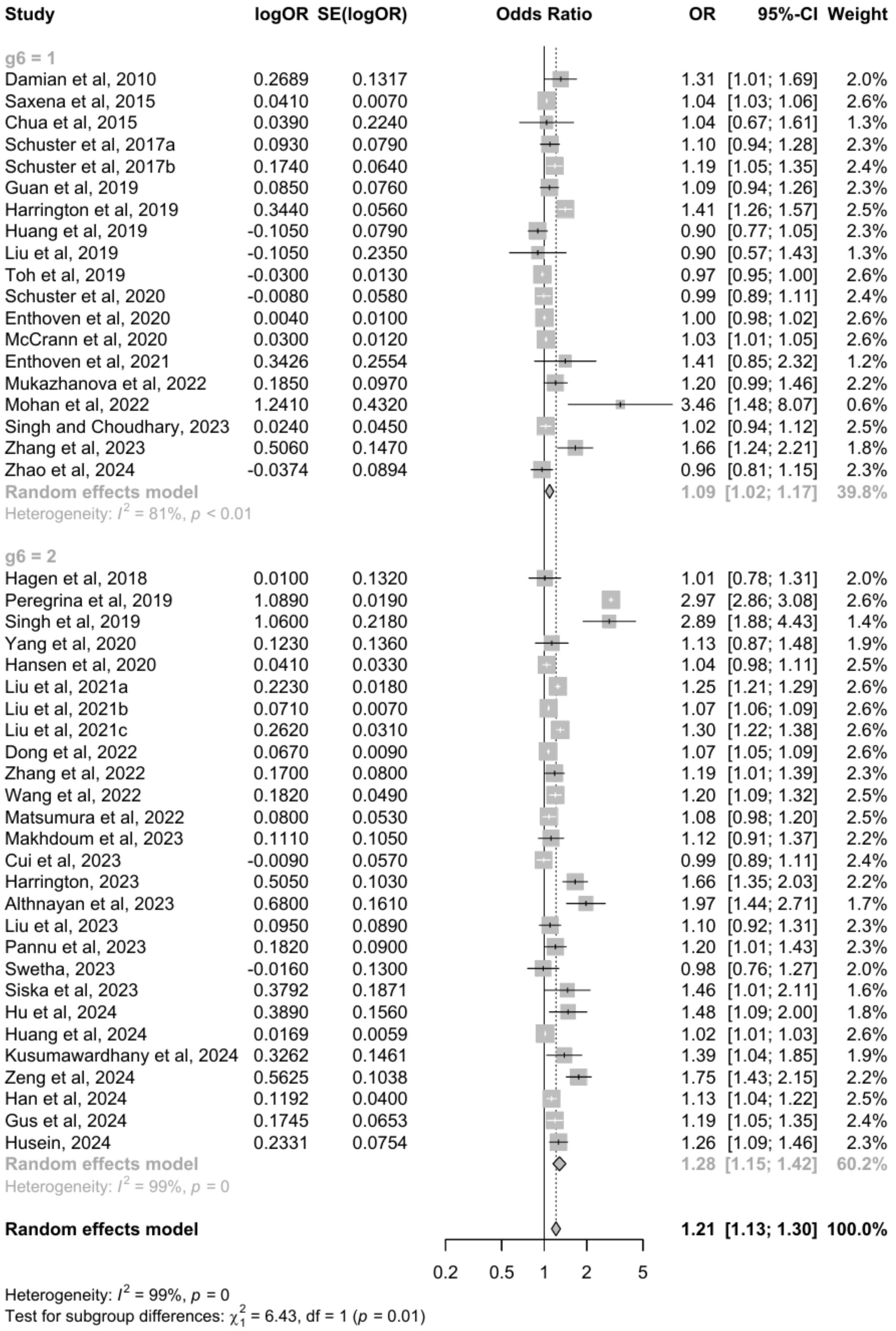

eFigure 7. Sensitivity analysis including only studies with myopia confirmed by cycloplegic refraction. OR = odds ratio; CI = confidence interval.

© 2025 Ha A et al. *JAMA Network Open*.

Study	logOR	SE(logOR)	Odds Ratio	OR	95%-CI	Weight
g5 = 1						
Damian et al, 2010	0.2689	0.1317		1.31	[1.01; 1.69]	2.0%
Saxena et al, 2015	0.0410	0.0070		1.04	[1.03; 1.06]	2.6%
Chua et al, 2015	0.0390	0.2240		1.04	[0.67; 1.61]	1.3%
Hagen et al, 2018	0.0100	0.1320		1.01	[0.78; 1.31]	2.0%
Guan et al, 2019	0.0850	0.0760		1.09	[0.94; 1.26]	2.3%
Harrington et al, 2019	0.3440	0.0560		1.41	[1.26; 1.57]	2.5%
Singh et al, 2019	1.0600	0.2180		2.89	[1.88; 4.43]	1.4%
Liu et al, 2019	-0.1050	0.2350		0.90	[0.57; 1.43]	1.3%
Enthoven et al, 2020	0.0040	0.0100		1.00	[0.98; 1.02]	2.6%
Enthoven et al, 2021	0.3426	0.2554		1.41	[0.85; 2.32]	1.2%
Mukazhanova et al, 2022	0.1850	0.0970		1.20	[0.99; 1.46]	2.2%
Wang et al, 2022	0.1820	0.0490		1.20	[1.09; 1.32]	2.5%
Mohan et al, 2022	1.2410	0.4320		3.46	[1.48; 8.07]	0.6%
Harrington, 2023	0.5050	0.1030		1.66	[1.35; 2.03]	2.2%
Althnayan et al, 2023	0.6800	0.1610		1.97	[1.44; 2.71]	1.7%
Liu et al, 2023	0.0950	0.0890		1.10	[0.92; 1.31]	2.3%
Pannu et al, 2023	0.1820	0.0900		1.20	[1.01; 1.43]	2.3%
Singh and Choudhary, 2023	0.0240	0.0450		1.02	[0.94; 1.12]	2.5%
Gus et al, 2024	0.1745	0.0653		1.19	[1.05; 1.35]	2.4%
Husein, 2024	0.2331	0.0754		1.26	[1.09; 1.46]	2.3%
**Random effects model**				**1.24**	**[1.13; 1.36]**	**40.0%**
Heterogeneity: $I^2 = 86\%$, $p < 0.01$						

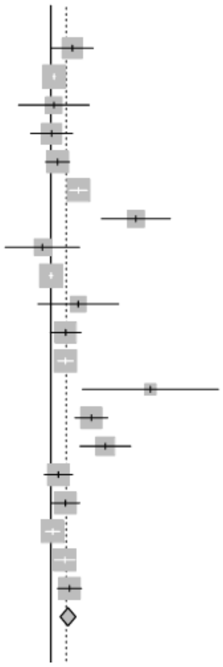

© 2025 Ha A et al. *JAMA Network Open*.

eFigure 8. Sensitivity analysis excluding studies with fewer than 500 participants. OR = odds ratio; CI = confidence interval.

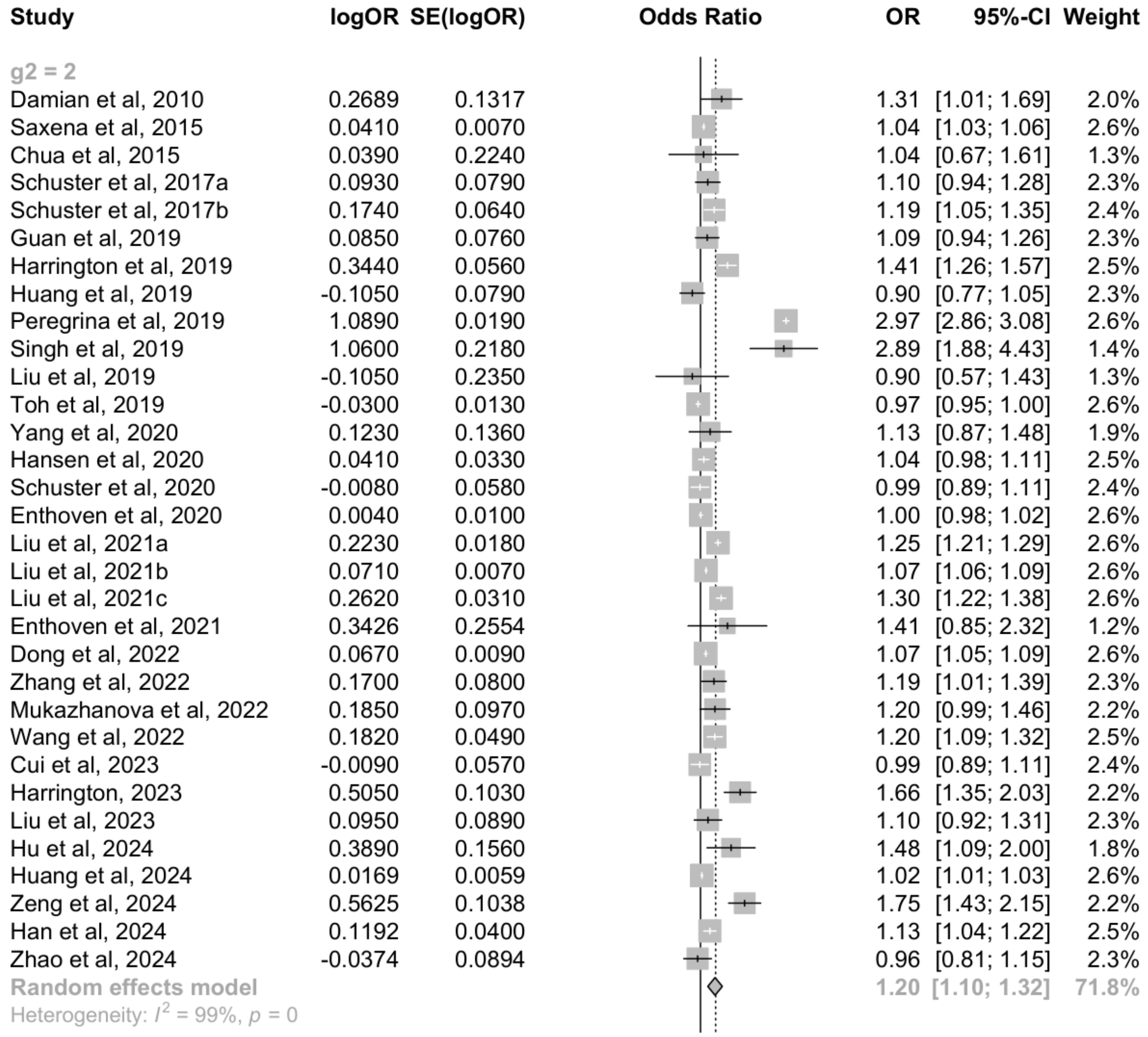

© 2025 Ha A et al. *JAMA Network Open*.

eFigure 9. Sensitivity analysis excluding studies with unadjusted data. OR = odds ratio; CI = confidence interval.

g9 = 1				
Saxena et al, 2015	0.0410	0.0070	1.04 [1.03; 1.06]	2.6%
Chua et al, 2015	0.0390	0.2240	1.04 [0.67; 1.61]	1.3%
Schuster et al, 2017a	0.0930	0.0790	1.10 [0.94; 1.28]	2.3%
Schuster et al, 2017b	0.1740	0.0640	1.19 [1.05; 1.35]	2.4%
Hagen et al, 2018	0.0100	0.1320	1.01 [0.78; 1.31]	2.0%
Guan et al, 2019	0.0850	0.0760	1.09 [0.94; 1.26]	2.3%
Harrington et al, 2019	0.3440	0.0560	1.41 [1.26; 1.57]	2.5%
Singh et al, 2019	1.0600	0.2180	2.89 [1.88; 4.43]	1.4%
Liu et al, 2019	-0.1050	0.2350	0.90 [0.57; 1.43]	1.3%
Toh et al, 2019	-0.0300	0.0130	0.97 [0.95; 1.00]	2.6%
Yang et al, 2020	0.1230	0.1360	1.13 [0.87; 1.48]	1.9%
Hansen et al, 2020	0.0410	0.0330	1.04 [0.98; 1.11]	2.5%
Schuster et al, 2020	-0.0080	0.0580	0.99 [0.89; 1.11]	2.4%
McCrann et al, 2020	0.0300	0.0120	1.03 [1.01; 1.05]	2.6%
Liu et al, 2021a	0.2230	0.0180	1.25 [1.21; 1.29]	2.6%
Liu et al, 2021b	0.0710	0.0070	1.07 [1.06; 1.09]	2.6%
Liu et al, 2021c	0.2620	0.0310	1.30 [1.22; 1.38]	2.6%
Enthoven et al, 2021	0.3426	0.2554	1.41 [0.85; 2.32]	1.2%
Dong et al, 2022	0.0670	0.0090	1.07 [1.05; 1.09]	2.6%
Zhang et al, 2022	0.1700	0.0800	1.19 [1.01; 1.39]	2.3%
Wang et al, 2022	0.1820	0.0490	1.20 [1.09; 1.32]	2.5%
Mohan et al, 2022	1.2410	0.4320	3.46 [1.48; 8.07]	0.6%
Makhdoum et al, 2023	0.1110	0.1050	1.12 [0.91; 1.37]	2.2%
Cui et al, 2023	-0.0090	0.0570	0.99 [0.89; 1.11]	2.4%
Harrington, 2023	0.5050	0.1030	1.66 [1.35; 2.03]	2.2%
Swetha, 2023	-0.0160	0.1300	0.98 [0.76; 1.27]	2.0%
Zhang et al, 2023	0.5060	0.1470	1.66 [1.24; 2.21]	1.8%
Siska et al, 2023	0.3792	0.1871	1.46 [1.01; 2.11]	1.6%
Hu et al, 2024	0.3890	0.1560	1.48 [1.09; 2.00]	1.8%
Huang et al, 2024	0.0169	0.0059	1.02 [1.01; 1.03]	2.6%
Zeng et al, 2024	0.5625	0.1038	1.75 [1.43; 2.15]	2.2%
Han et al, 2024	0.1192	0.0400	1.13 [1.04; 1.22]	2.5%
Gus et al, 2024	0.1745	0.0653	1.19 [1.05; 1.35]	2.4%
Zhao et al, 2024	-0.0374	0.0894	0.96 [0.81; 1.15]	2.3%
**Random effects model**			**1.17 [1.10; 1.24]**	**72.9%**
Heterogeneity: $I^2 = 91\%$, $p < 0.01$				

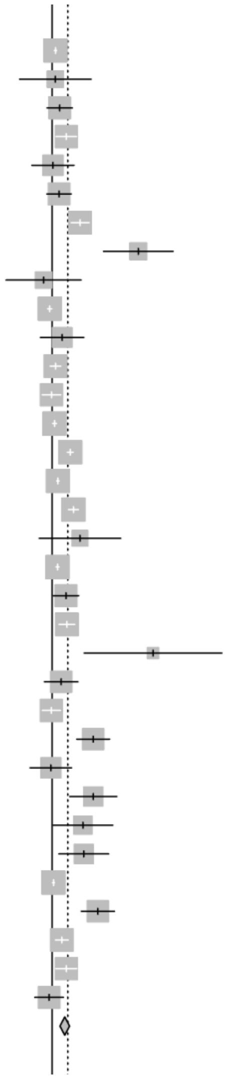

eFigure 10. Sensitivity analysis including only studies with screen time exposure limited to smartphone use. OR = odds ratio; CI = confidence interval.

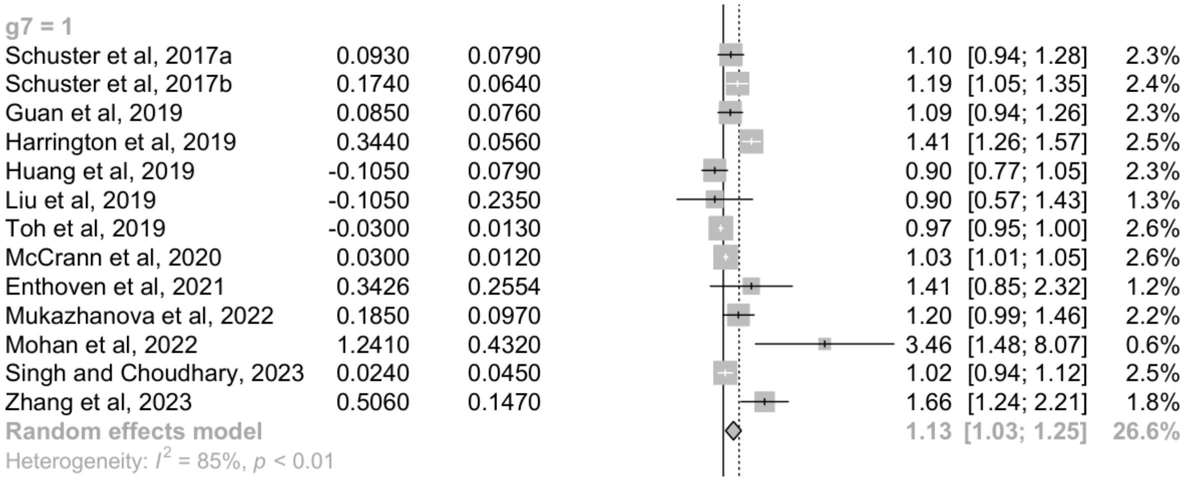

© 2025 Ha A et al. *JAMA Network Open*.

eFigure 11. Association between Digital Screen Time and Logarithm of Odds Ratio for Myopia Risk. Each circle represents the individual study, the solid line indicates risk estimates, and the shaded areas represent 95% confidence intervals.

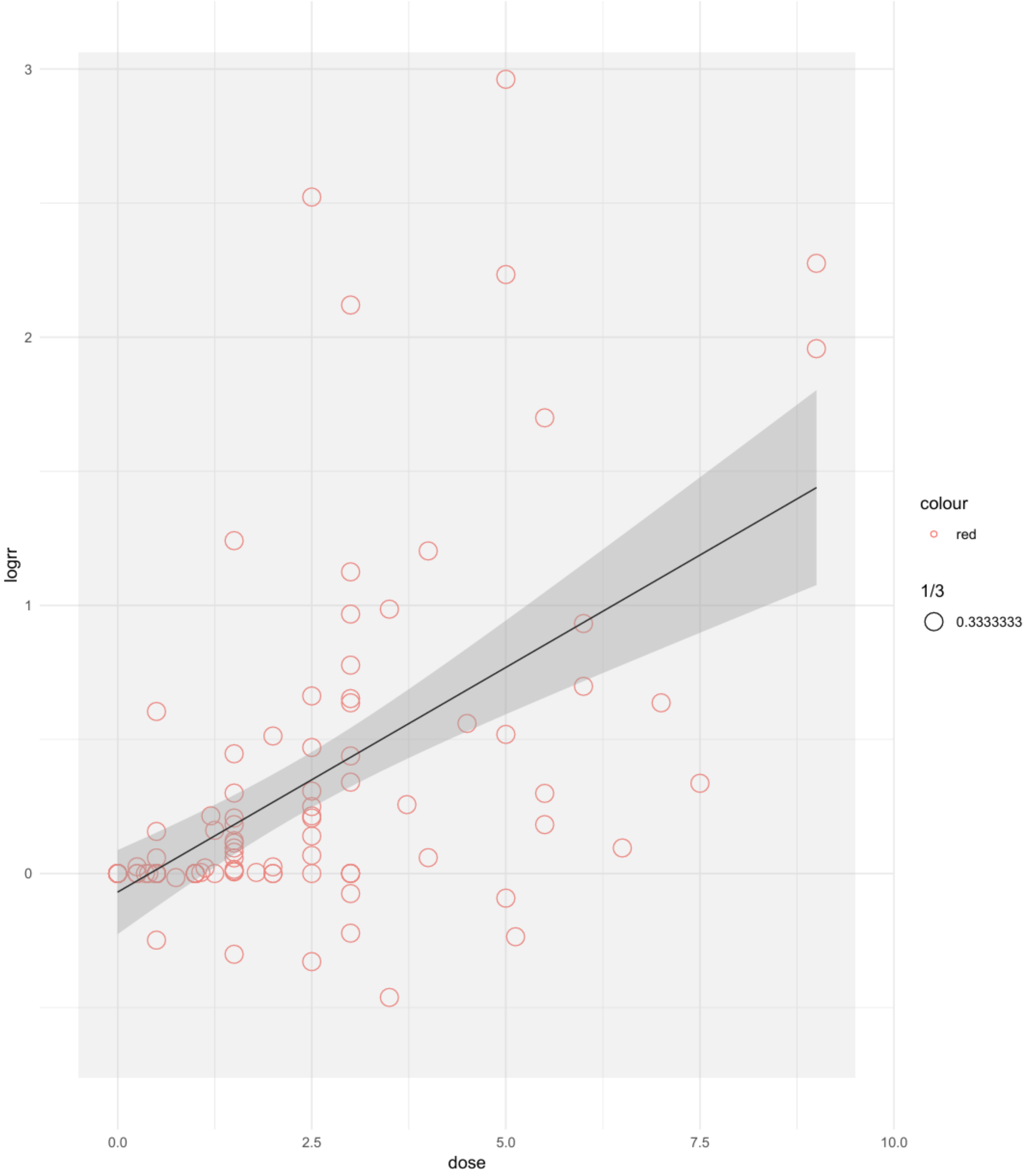

© 2025 Ha A et al. *JAMA Network Open.*

eFigure 12. Sensitivity Analyses of Dose-Outcome Curve. A. flexible restricted cubic splines with knots positioned at 10th, 30th, 70th, and 90th percentiles; **B.** knots placed at 15th, 40th, 60th, and 85th percentiles; **C.** including only studies where myopia was confirmed by cycloplegic refraction; **D.** excluding studies with fewer than 500 participants; **E.** including studies only participants under 19 years of age

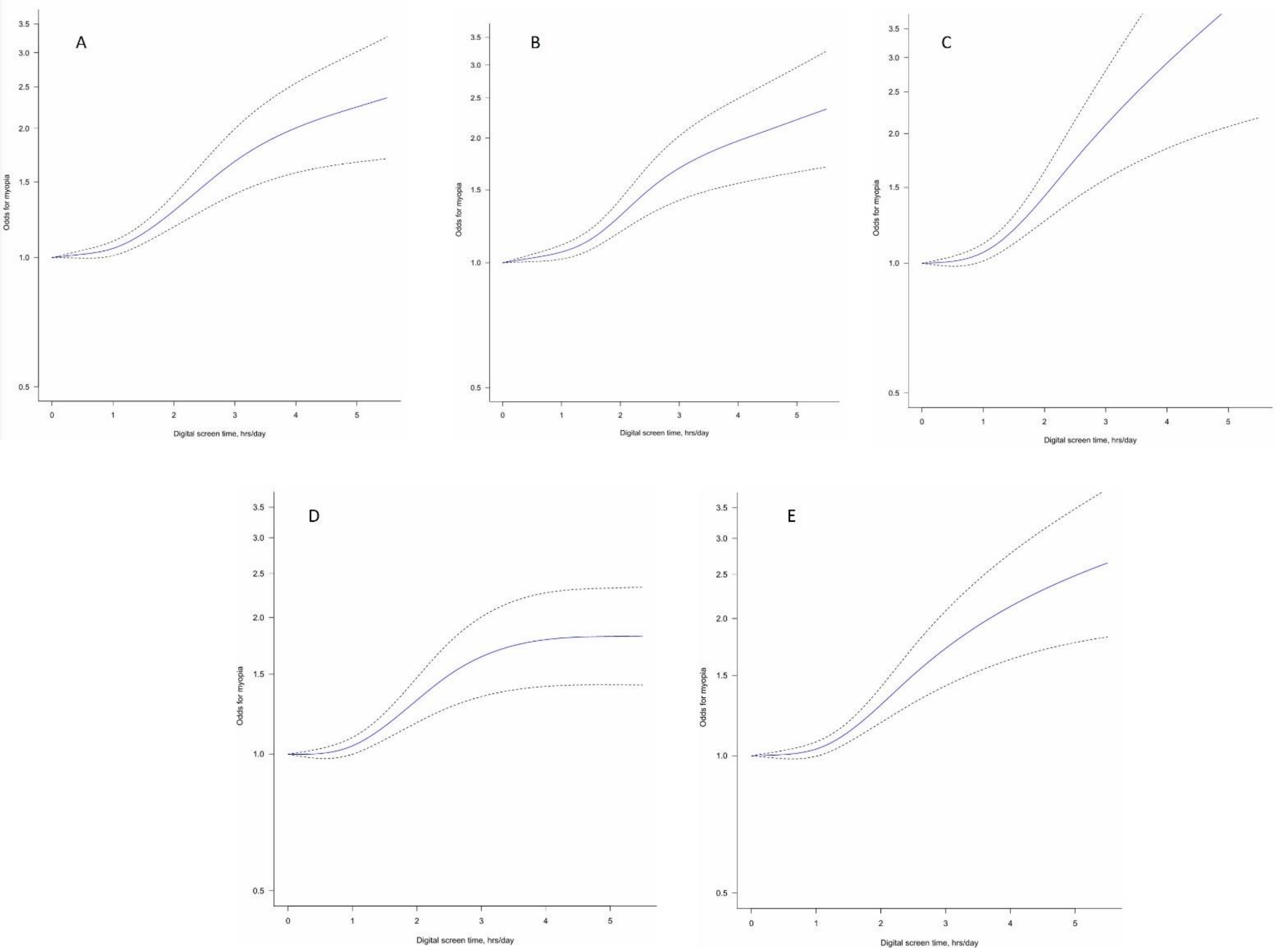

© 2025 Ha A et al. *JAMA Network Open*.

eFigure 13. Contour-enhanced funnel plots for the primary outcome (45 studies)

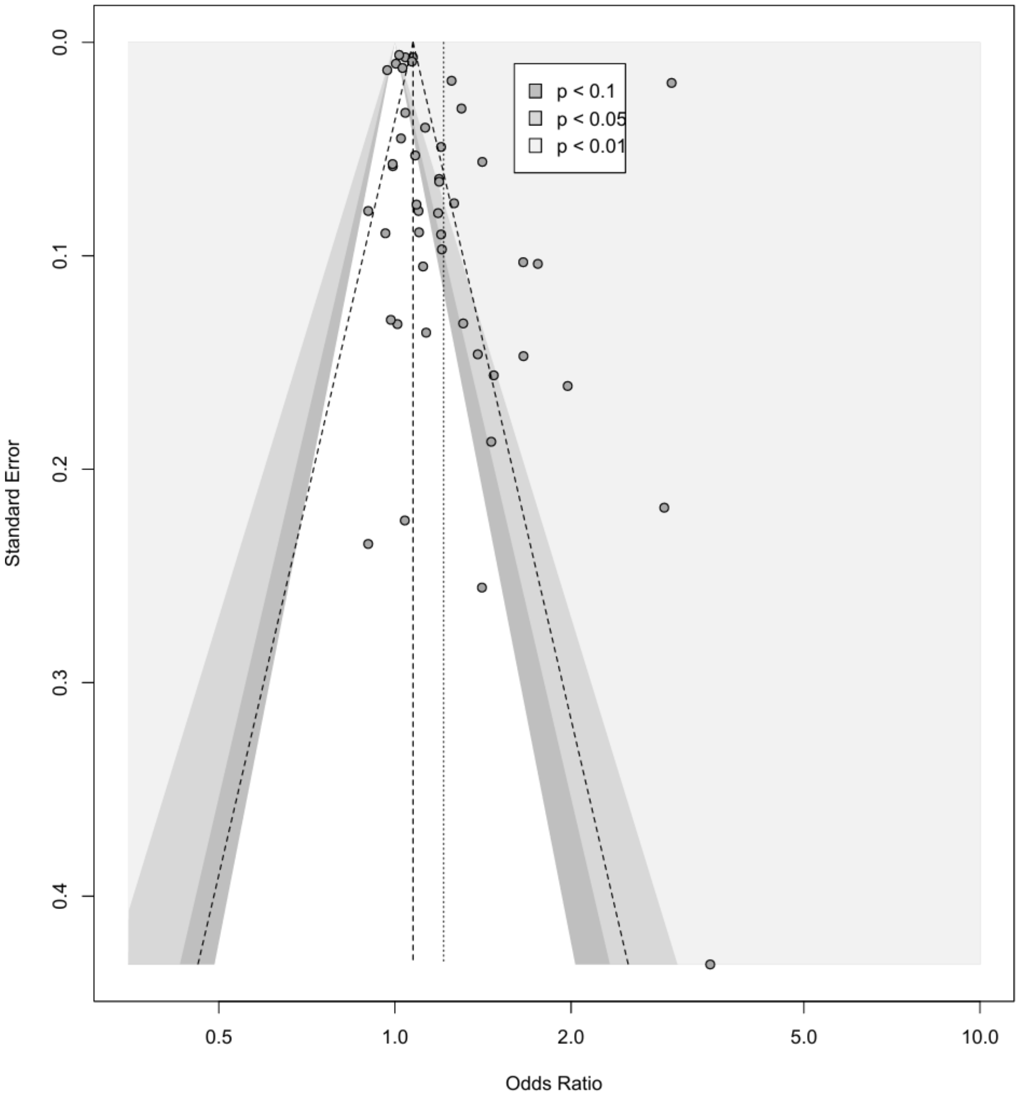

Supplement: Supplement 1. — eAppendix 1. Search Term eAppendix 2. Newcastle-Ottawa Scale Assessment Scale eMethods. Methods for Converting Effect Sizes eFigure 1. Flow Diagram of Selection Process for Inclusion of Studies in Meta-Analysis eAppendix 3. References to Included Studies eAppendix 4. List of Articles Excluded at Full-Text Screening Stage\ eTable 1. Risk of Bias Assessment for Individual Studies eFigure 2. Subgroup Analysis Based on Myopia-Related Outcomes eFigure 3. Subgroup Analysis Based on Study Design eFigure 4. Subgroup Analysis Based on Participant Age eFigure 5. Subgroup Analysis Comparing Studies Conducted in Asia to Those Conducted Elsewhere eFigure 6. Subgroup Analysis Comparing Studies With Combined Device Analysis vs. Individual Device Analysis eFigure 7. Sensitivity Analysis Including Only Studies With Myopia Confirmed by Cycloplegic Refraction eFigure 8. Sensitivity Analysis Excluding Studies With Fewer Than 500 Participants eFigure 9. Sensitivity Analysis Excluding Studies With Unadjusted Data eFigure 10. Sensitivity Analysis Including Only Studies With Screen Time Exposure Limited to Smartphone Use eFigure 11. Association Between Digital Screen Time and Logarithm of Odds Ratio for Myopia Risk eFigure 12. Sensitivity Analyses of Dose-Outcome Curve eFigure 13. Contour-Enhanced Funnel Plots for the Primary Outcome (45 Studies) [file jamanetwopen-e2460026-s001.pdf]
