# Supplementary material for: Digital Screen Time and Myopia: A Systematic Review and Dose-Response Meta-Analysis
Source: JAMA Netw Open. 2025 Feb 21;8(2):e2460026. doi: 10.1001/jamanetworkopen.2024.60026 (PMC11846013; doi:10.1001/jamanetworkopen.2024.60026)
Supplement: Supplement 2. — Data Sharing Statement [file jamanetwopen-e2460026-s002.pdf]

Data Sharing Statement

Ha. Digital Screen Time and Myopia. *JAMA Netw Open*. Published February 21, 2025.
doi:10.1001/jamanetworkopen.2024.60026

Data
Data available: No

Additional Information
Explanation for why data not available: All articles in this manuscript are available from PubMed, Embase, CINAHL, and The Cochrane Library. The analysis codes are available from the corresponding author on reasonable request.
